# Mediation of the association between education and dementia by occupational complexity, income, health behaviours and health outcomes

**DOI:** 10.1186/s12888-025-06619-4

**Published:** 2025-02-25

**Authors:** Katherine Taylor, Louise Marston, Naaheed Mukadam

**Affiliations:** 1 Division of Biosciences, Medical Sciences Building, University College London, Gower Street, London, WC1E 6BT U.K.; 2 Department of Primary Care and Population Health, University College London, Rowland Hill Street, London, NW3 2PF U.K.; 3 UCL Division of Psychiatry, University College London, 1st Floor Maple House, 149 Tottenham Court Road, London, W1T 7NF U.K.; 4 Camden and Islington NHS Foundation Trust, St Pancras Hospital, 4 St Pancras Way, London, NW1 0PE U.K.

**Keywords:** Causal mediation analysis, Cognitive reserve, UK biobank

## Abstract

**Background:**

Many studies observe a negative association between education and all-cause dementia, however, little is known about how the association develops. Current evidence regarding mediatory factors is limited, conflicted and suggests a complex relationship. In this study we used UK Biobank data to determine to what extent multiple factors mediate the association.

**Methods:**

Data was sourced from UK Biobank and multiple imputation used to replace missing values. Education was measured at baseline assessment and dementia diagnosis determined from self-report or linked healthcare records. Five potential mediators were considered: Social isolation, income and occupational complexity were measured at baseline and health behaviour and health outcome scores derived. Logistic regression was used to examine the association between education and dementia with adjustment for potential mediators. Causal mediation analysis was then performed to determine the proportion of the dementia education association occurring via each mediatory pathway.

**Results:**

In a sample of 384,284 participants, higher level of education was associated with reduced odds of dementia. When considered as a confounder, occupational complexity almost fully attenuated the association (OR: 0.94, CI: 0.89–0.99) and was found to mediate 73% of the association. Average income, health outcomes and health behaviours also acted as mediators, explaining 10%, 27% and 35% of the relationship respectively.

**Conclusions:**

Occupational complexity mediates a large proportion of the association between education and dementia. Intervention to improve access to cognitively stimulating work and leisure activities, particularly to those who are less educated, may reduce the number of people in the UK living with dementia.

**Supplementary Information:**

The online version contains supplementary material available at 10.1186/s12888-025-06619-4.

## Background

In 2024, one in every three people born will develop dementia. By 2025, it is predicted to cost the UK £47 billion [1]. Despite the high societal impact, we have limited disease modifying treatments. Therefore, research into potentially modifiable risk factors is vital. Current evidence finds education to be protective against dementia. Decreases in both its prevalence and incidence are observed in association with higher levels of education [2–5]. Education appears to play a pivotal role in the build-up of cognitive reserve, which is maintained until later life [6, 7]. However, it is unclear to what extent this build-up of cognitive reserve directly influences dementia risk and whether other factors mediate the association [2–5, 8–10].

### Potential pathways

#### Health outcomes and behaviours

Evidence looking into the role of health behaviours and health outcomes as mediators of the association between education and dementia is mixed and intertwined. It is hypothesised that those who have a higher level of education are more likely to adopt certain health behaviours, such as not smoking, resulting in fewer health conditions and lower risk of dementia [11–15]. One study observed the association between education and dementia persists upon adjustment for cardiovascular risk factors [8]. Another found that 17% of the association was mediated by a cardiovascular health score that included smoking, physical activity, healthy diet, and cholesterol [16]. Additionally, it was observed that the presence of hypertension partially mediated the association between education and amyloid-beta negative subcortical vascular dementia [17]. However, studies were inconsistent with another finding that history of a stroke and physical activity level did not have a mediatory effect [18].

Education is often used as a proxy measure of socioeconomic status because it is strongly associated with childhood socio-economic conditions [19]. Prior work investigating the association between socioeconomic status (SES) and dementia considered the potential of a poly-environmental risk score (LIBRA) comprising health conditions and protective factors to mediate the association. When education was incorporated into it as a measure of SES, LIBRA score mediated 12.7% of the relationship between SES and cognitive functioning [20]. Further work found no direct associations between education and dementia but observed multiple indirect pathways, one through LIBRA score and one via wealth and LIBRA score [21]. These findings highlight the complex relationships between mid-life health, SES, and dementia.

#### Occupational complexity

Work investigating the potential for mid-life occupation to mediate the relationship between education and dementia is limited. The cognitive reserve hypothesis refers to the ability to tolerate disease related pathology without clinical symptoms [3]. It is assumed that cognitive activity, such as education or more complex occupation, shapes neural activity, building up cognitive reserve [2]. One study found that low education is associated with Alzheimer’s disease incidence independent of occupation-based SES, regardless of the duration on low-SES occupations and adult socioeconomic mobility pattern [22]. This suggests the association between education and dementia occurs independent of occupational SES. Contrary to this, two mediation studies have found occupational complexity to partially mediate the relationship. Causal mediation analysis estimated 28% of the effect of education on dementia free survival time was mediated by occupational complexity [23]. Another found that 11-12% of the protective effect of education was mediated by occupational complexity, though the pattern and magnitude of the mediating effect differed between white men and other race-gender groups [24].

#### Average income

To our knowledge, no studies have specifically investigated the role of income as a mediator of the relationship between education and dementia. However, the indirect pathway found in prior work implicates income as one [21]. Those with higher education are likely to have a higher income and multiple studies have found that those with higher income have a lower risk of dementia [25–27]. Therefore, it is possible that income mediates the association.

#### Social isolation

One study considered the mediatory effect of social isolation on the association between education and dementia, but no significant effects were observed [18]. Lower social contact has been shown in numerous studies to increase an individual’s risk of developing dementia but associations between education and social isolation are more complex [18, 28, 29]. They do not appear to be monotonic across education levels. However, those who were more educated were found to have a significantly lower probability of feeling lonely and were more likely to have someone to confide in [30]. Therefore, it is possible that social isolation could act as a mediator.

### Current work

In summary, literature regarding mediation of the relationship between education and dementia is limited, conflicting and suggests a complex relationship between education and mid-life factors. Health outcomes and health behaviours are rarely considered as separate entities therefore, we lack clarity on how education acts through midlife health. Research looking at occupational complexity implicates it as a likely mediator, but further evidence is required to estimate its relative importance in comparison with other potential pathways. Very few studies investigate the roles of social isolation and income.

In this study we aim to build on previous work using UK Biobank data to carry out a mediation analysis of the association between education and dementia considering health behaviours, health outcomes, occupational complexity, average income, and social isolation.

## Methods

### Data source

This research has been conducted using the UK Biobank Resource (UKB; www.ukbiobank.ac.uk). Ethical approval was gained from the National Research Ethics Service Committee North West. Details regarding the sample and collection methods have been described elsewhere [31]. We obtained ethical approval from UKB prior to acquiring data.

### Measures

#### Main exposure– education

Education was measured through a baseline touchscreen questionnaire (ranging from none to college or university degree) and was categorised into education up to age 16 and education post 16. Education was dichotomised as in prior studies, when education was divided into more than two categories, there was reduced power for observing the impact of category of education closest to the reference group [4].

#### Main outcome– dementia

A clinical diagnosis of “all-cause dementia” was obtained from self-report, or from linked electronic hospital, primary care, or mortality records. The validity of this approach has previously been demonstrated [32].

#### Confounders

We included: self-reported sex (male/female), age at baseline, ethnicity (white/other), Townsend deprivation index score (calculated based on residential postcode) and depression.

#### Mediators

We selected mediators based on previous literature [18, 21, 23, 33].

#### Health behaviours

A health behaviour score was comprised by collating measures of self-reported diet, smoking, physical activity, and alcohol intake. A binary diet score was created based on consumption of at least 4 of the 7 commonly eaten food groups in line with priorities for cardiometabolic health [34]. A binary physical activity measure was derived based on WHO guidelines of 600 Metabolic Equivalent Task min/week [35]. Smoking was categorised into current smoker and current non-smoker. Alcohol consumption was categorised based on drinking above recommended limits (> 21 units/week) or not [36]. Lifestyle scores were collated, with a higher score indicating less favourable lifestyle behaviours. It was then categorised as follows: 0 and 1 as favourable and 2 to 4 as unfavourable. This dichotomisation was chosen to get a broader picture of an individual’s lifestyle distinguishing between those who have largely favourable lifestyle behaviours and those who had multiple health behaviours which fall outside of recommended guidelines.

#### Health outcomes

A health outcome score was derived, with a higher score indicating worse health. The following binary measures were collated to give the total score: hypertension defined as anyone using antihypertensive medications or who had a baseline blood pressure > 140/90mmHg; diabetes based on self-reported diagnostic status or reported use of insulin; obesity was based on BMI at baseline (BMI ≥ 30 considered obese) and depression was defined as those using antidepressants or who reported seeking help for depression from a GP or psychiatrist. Scores for all health conditions were added the give a total health score. Total health score was categorised into good/excellent health (score of 0 or 1) and intermediate/poor health (score of 2 or 3). This dichotomisation was chosen as 30% of the complete case sample had one health condition. These individuals were included in the good/excellent health category as we determined that having two or more conditions was a better indicator of overall poor health.

#### Social isolation

A binary measure of social isolation was created using responses from a baseline touchscreen questionnaire. Those who had less than almost daily social contact with friends, family and who lived alone were considered socially isolated and those with more frequent social contact as not socially isolated [37, 38].

#### Income

Average household income was self-reported at baseline assessment (values ranging from < 18,000 to > 100,000) and categorised into < 31,000 and > 30,999. Income was categorised around the average income, 45% of the population had an income <£31,000 and 55% had an income of > £30,999.

#### Occupational complexity

Occupational complexity was derived from job Standard Occupational Code (SOC) measured at baseline assessment [39]. It was categorised into managerial and professional occupations and non-managerial and professional occupations. Retired people were classified according to their last occupation. Occupation was dichotomised in this way as in complete case data, 57.5% of the sample were classified as having managerial or professional occupations.

### Exclusion criteria

Those with prevalent dementia, or dementia diagnosed up to one year after baseline were excluded. UK Biobank is a relatively young cohort so one year was chosen to maximise follow-up time. Those who withdrew consent to inclusion were excluded, as were those aged under 50 as they were unlikely to have developed dementia during the follow-up period.

### Statistical analysis

We investigated patterns within missing data to determine whether we can assume data was missing at random. Following this, multiple imputation by chained equations was used to generate values for all missing data. 20 imputed datasets were generated [40] and these were used for our primary analysis. Sensitivity analysis was performed by repeating analysis on complete case data. Sample characteristics were described, and bivariate associations between education and variables examined.

All analysis were carried out in R studio, version 4.2.2.

#### Logistic regression

We used logistic regression [41] with education as exposure and dementia as outcome. All models were adjusted for age, sex, and ethnicity. Mediators were added to the model in the following order: health outcomes, social isolation, health behaviours, income and occupational complexity. Income and occupational complexity were not adjusted for in the same model to avoid collinearity.

#### Mediation analysis

We evaluated the association between education and dementia on the consideration of potential mediations: health outcomes, social isolation, average income, health behaviours, and occupational complexity.

Causal mediation analysis was used as traditional mediation analysis methods do not generalise well to models with binary variables. Non-collapsibility means that the scale of logistic regressions used in traditional mediation analysis are different and so cannot be compared. The difference-in-coefficient approach conflates the indirect effect and non-collapsibility and, in some cases, can falsely indicate the presence of an indirect effect. Causal mediation analysis overcomes these issues by using a potential outcomes framework, providing general definitions of causal direct and indirect effects that can be applied to any mediation model [42].

**Fig. 1:**
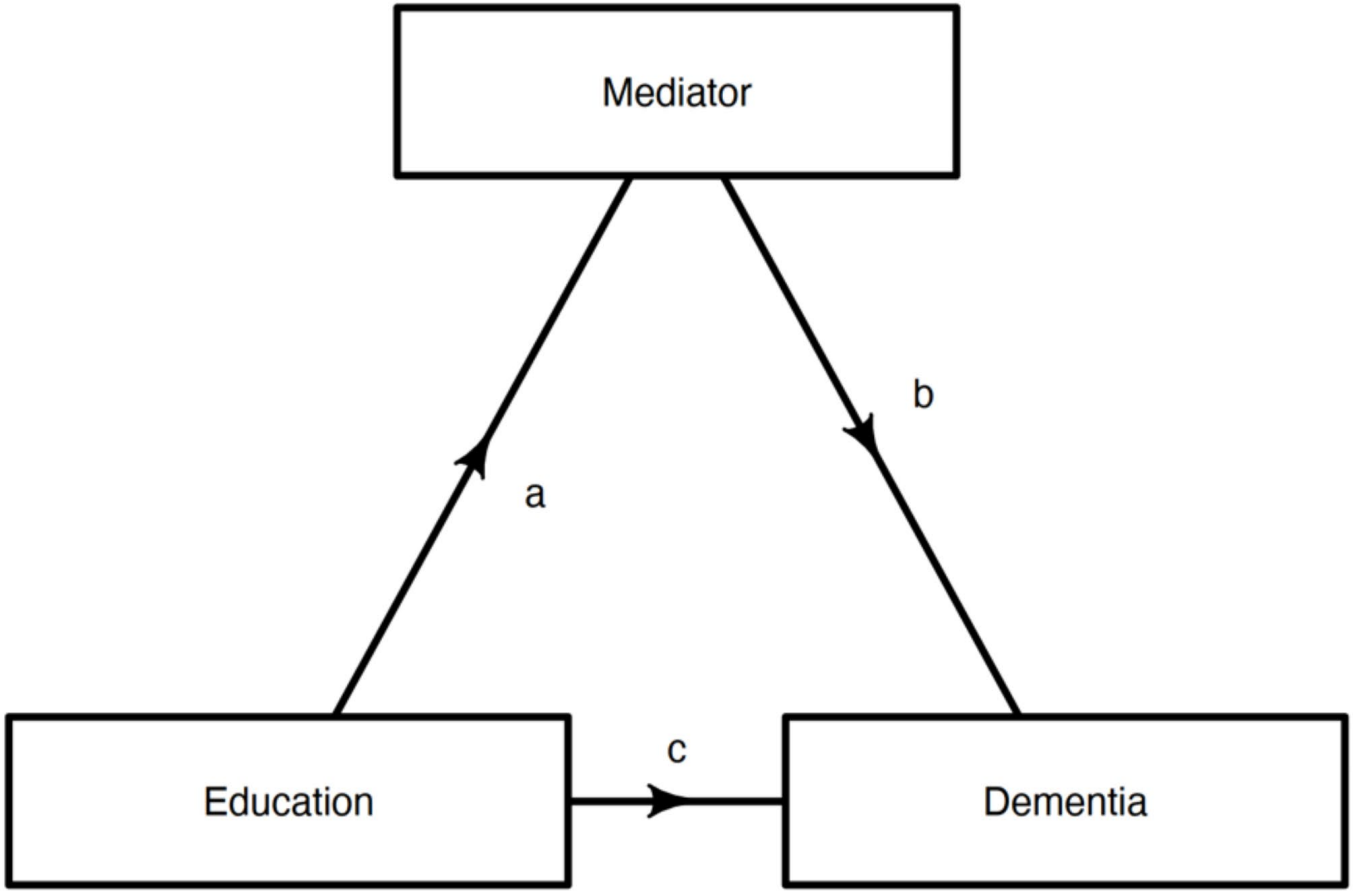
Model displaying the hypothesised relationships between education, dementia and mediating variables

Figure 1 displays our hypothesised model. The relation between education and the potential mediator is denoted by a, b represents the relation of the potential mediator to dementia and c the relation of education to dementia, adjusted for the mediator. To address causal assumptions relating to unmeasured confounding, we drew directed acyclic graphs (DAGs) based on existing literature to identify confounders of each pathway [33]. In each model, all associations are adjusted for age, sex and ethnicity. The following mediator-outcome confounders were adjusted for: health behaviour, average income and occupational complexity in the health outcome mediation model; health outcomes, average income and occupational complexity in the health behaviour mediation model; health behaviour and health outcomes in the average income mediation model; health outcomes and health behaviour in the occupational complexity mediation model and depression in the social isolation mediation model. Occupation was not adjusted for in the income mediation model and vice-versa to avoid collinearity.

Causal mediation analysis was performed using the mediate package which uses a simulation-based approach to estimate potential mediator and outcome values [43]. 95% CI and β coefficients for the direct, causal mediation and total effects were calculated. The direct effect refers to the association between education and dementia, independent of the mediator (Fig. 1, path c). The causal mediation effect represents how much of the association between education and dementia is explained by the mediator (Fig. 1, paths a and b). The total effect is the direct effect plus the causal mediation effect. The proportion of the association mediated by each mediator was also calculated by dividing the average causal mediation effect by the total effect. We assessed the confidence intervals of effect sizes and proportions using 1000 nonparametric bootstrap simulations. Additionally, we tested for exposure-mediator interactions by incorporating an interaction term into the model. To evaluate evidence for an interaction we tested for statistical significance and compared effect sizes in models with and without the interaction term, defining a significant interaction as one which changed the average proportion mediated by more than 10% [44].

## Results

**Fig. 2:**
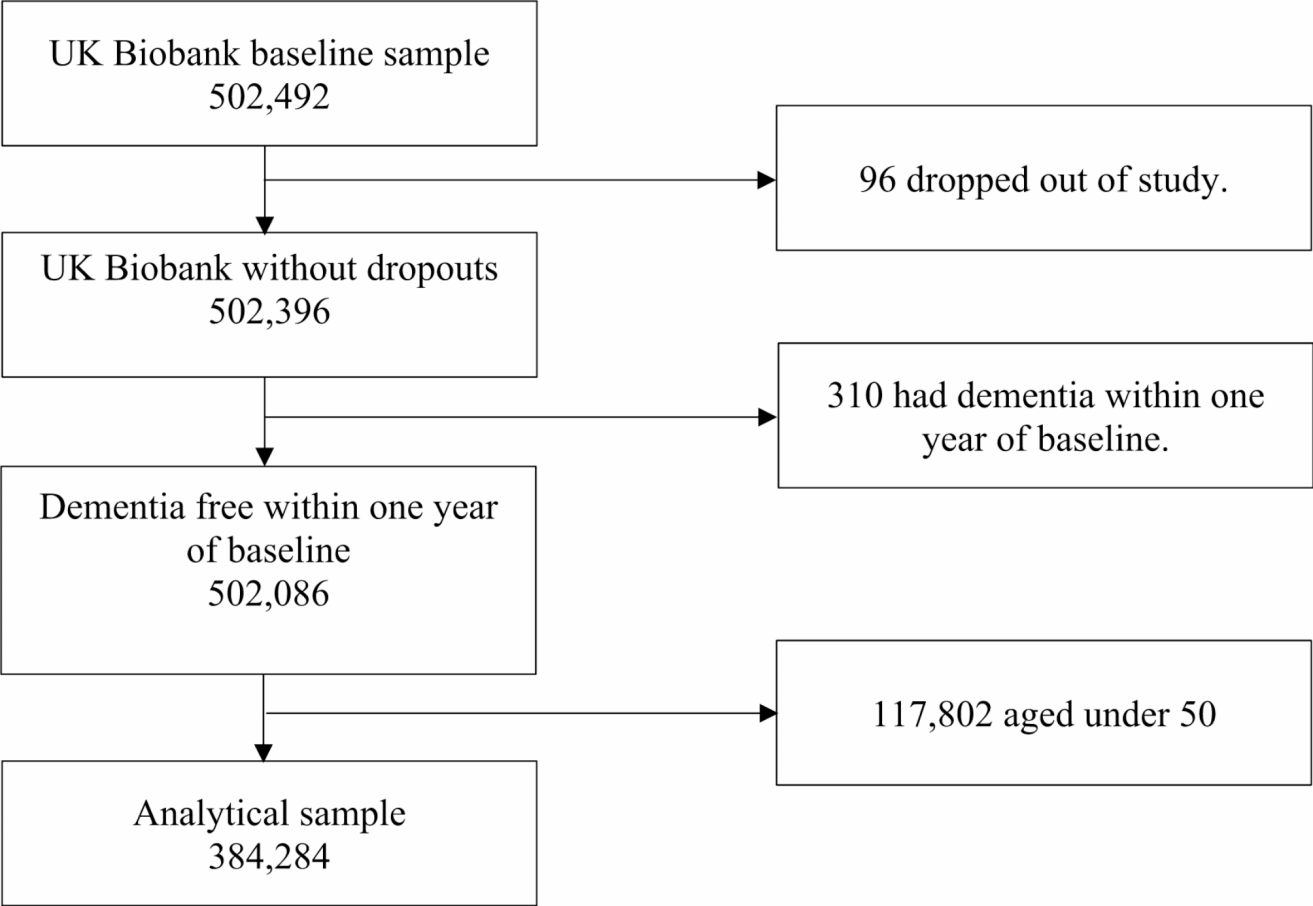
Strobe diagram of analytical sample selection from the baseline UK Biobank sample

Baseline assessments took place between March 2006 and October 2010. UKB had a baseline sample of 502,492 participants. 96 participants dropped out of the study and 310 participants were excluded from the study as they had either prevalent dementia or dementia diagnosed within one year of the baseline. 117,802 individuals were excluded as they were aged under 50. This resulted in a total sample of 384,284 (Fig. 2). The mean age at baseline assessment was 60 (SE:0.01) and data on dementia incidence was collected until December 2021.

Exploration of associations within the dataset supported assumptions that the data was missing at random and therefore the dataset generated through multiple imputation was used in primary analysis. Details of baseline characteristics in the multiple imputed sample are presented in Table 1. Amongst those with dementia, 59% had education to age 16 compared to 41% amongst those without dementia. Those with higher levels of education were on average younger, more likely to be male and less likely to be Caucasian compared to those with less education.

**Table 1:** Descriptive analysis of imputed data

	Education after 16	Education up 16
**Dementia**		
No dementia	55%	45%
Dementia	41%	59%
**Sex**		
Female	54%	46%
Male	55%	45%
**Age**		
Mean (SE)	59.3 (0.01)	61.0 (0.01)
**Ethnicity**		
Caucasian	53%	47%
Not Caucasian	77%	23%
**Income**		
< 31,000	43%	57%
> 30,999	64%	36%
**Occupational Complexity**		
Managerial & professional	77%	23%
Non managerial or professional occupations	41%	60%
**Social Isolation**		
Not socially isolated	55%	46%
Socially isolated	53%	47%
**Health Outcomes**		
Good/Excellent Health	56%	44%
Intermediate/Poor health	45%	55%
**Health Behaviour**		
Favourable	57%	43%
Unfavourable	43%	57%
**Depression**		
No	54%	46%
Yes	59%	41%

The complete case sample had similar demographics to the imputed sample (Supplementary Table 1). 1.0% of the sample had a dementia diagnosis in the follow-up period, similar to 2% in the imputed sample. 38.6% of the sample had education up to age 16, 61.4% had education after the age of 16 (Supplementary Table 1).

### Logistic regression

In the minimally adjusted model, education was associated with reduced odds of dementia. Upon adjustment for each potential mediator, except social isolation, the association was attenuated. Adjusting for occupational complexity had the greatest impact. The reduced odds of dementia amongst those with education post 16 compared to those with education up to age 16 decreased from 20% (OR:0.80 95% CI:0.76 to 0.84) to 6% (OR:0.94 95% CI: 0.89 to 0.99) (Table 2).

**Table 2:** Logistic regression of the association between education and dementia upon adjustment for potential mediators

Model	OR	95% CI	
Adjusted for Age, Sex & Ethnicity	0.75	0.71,	0.78
+ Health Outcome	0.77	0.73,	0.80
+ Health Outcome & Social Isolation	0.77	0.73,	0.80
+ Health Outcome, Social Isolation & Health Behaviour	0.79	0.76,	0.83
+ Health Outcome, Social Isolation, Health Behaviour & Wealth	0.80	0.76,	0.84
+ Health Outcome, Social Isolation, Health Behaviour & Occupation	0.94	0.89,	0.99

### Mediation analysis

All results can be found in Table 3. No significant exposure-mediator interactions were observed (Supplementary Table 4).

**Table 3:** Summary effects from fully adjusted mediation models

	β	95% CI		Proportion Mediated (95% CI)
***Occupational Complexity as a mediator***				
Total Effect	0.0048	0.0039	0.0056	73% (73-74%)
Average Direct Effect	0.0013	0.0004	0.0022	
Average Causal Mediation Effect	0.0036	0.0032	0.0038	
***Income as a mediator***				
Total Effects	0.0045	0.0036	0.0054	10% (9-10%)
Average Direct Effect	0.0041	0.0032	0.0050	
Average Causal Mediation Effect	0.0004	0.0003	0.0006	
***Health Outcomes as a mediator***				
Total Effects	0.0016	0.0007	0.0025	27% (27-28%)
Average Direct Effect	0.0011	0.0002	0.0021	
Average Causal Mediation Effect	0.0004	0.0004	0.0005	
***Social Isolation as a mediator***				
Total Effects	0.0055	0.0047	0.0064	0% (0%-0%)
Average Direct Effect	0.0055	0.0047	0.0064	
Average Causal Mediation Effect	0.0000	0.0000	0.0000	
***Health Behaviours as a mediator***				
Total Effects	0.0018	0.0008	0.0027	35% (34-36%)
Direct Effects	0.0011	0.0002	0.0021	
Average Causal Mediation Effect	0.0006	0.0005	0.0007	

#### Occupational complexity

**Fig. 3:**
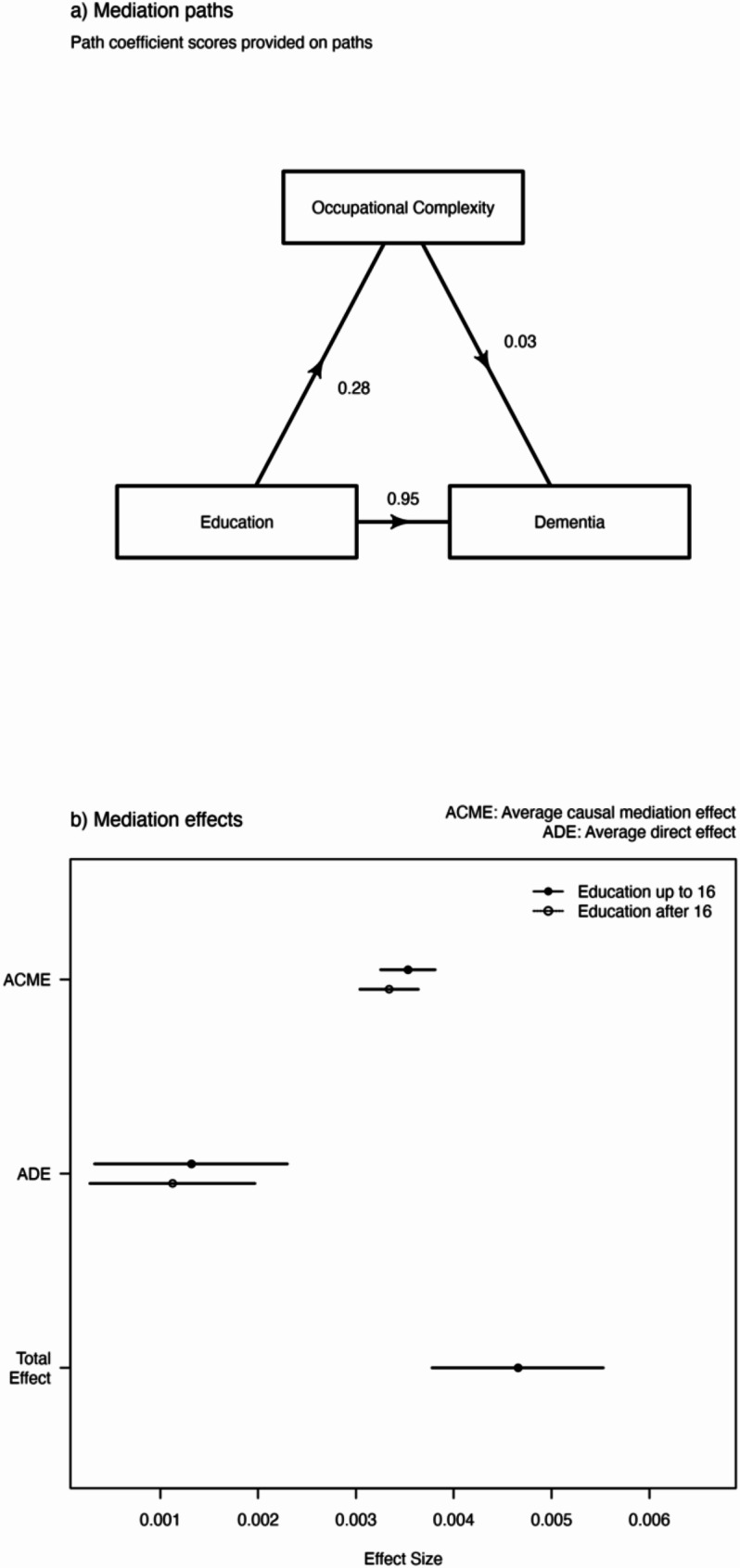
(**a**) Mediation paths of occupational complexity as a mediator. (**b**) Average causal mediation effect (ACME), average direct effect (ADE) for those with education up to and post the age of 16 and the total effect of education and occupational complexity on dementia

Occupational complexity has the largest mediatory effect, mediating approximately 73% (95% CI: 73-74%) of the association between education level and dementia. Those with lower education are predicted to have a lower occupational complexity which is associated with an increased risk of dementia (Fig. 3a). In causal mediation analysis, we find the average causal mediation effect (β: 0.0036, 95% CI: 0.0032–0.0038) to be greater than the average direct effect (β:0.0012, 95% CI: 0.0032–0.0037, Table 3; Fig. 3b).

#### Other mediators

Health behaviours, health outcomes and income are all observed to mediate the association between education and dementia. A higher level of education was associated with better health behaviour, health outcomes and income. All three mediators were negatively associated with dementia (Supplementary Fig. 1a, 2a, 3a). We find they mediate 35% (95% CI: 34-36%), 27% (95% CI: 27-28%) and 10% (95% CI: 9-10%) of the association respectively (Supplementary Fig. 1b, 2b, 3b). Social isolation was not observed to mediate the association (Table 3).

### Sensitivity analysis

In logistic regression performed on complete case data, the negative association between dementia and education remained significant upon adjustment for all covariates and potential mediators. Education displayed a greater protective effect within complete case data in comparison to imputed data, especially upon adjustment for occupational complexity. Within the complete case sample, those with more education have 24% lower odds (OR:0.86, 95% CI: 0.78–0.94) of developing dementia compared to those with less education in the fully adjusted model (Supplementary Table 2).

Occupational complexity remained the mediator with the biggest proportion mediated, though the size of the mediatory effect was smaller compared to estimates using imputed data (54%, 95% CI:39-76% compared to 73%, 95% CI: 73-74%). As with imputed data, income, health behaviours and health outcomes are observed to mediate the association, but not social isolation. In imputed data, health outcomes and health behaviours mediated a greater proportion of the association in comparison to income (mediating 27%, 35% and 10% respectively). However, the opposite is true in complete case data where income mediates 39% of the association but health outcomes and health behaviours only mediate 25% and 12% respectively (Supplementary Table 3).

## Discussion

### Key findings

As hypothesised this large prospective study found a negative relationship between education and all-cause dementia. Occupational complexity was observed to mediate 73% of the association and, upon consideration of occupational complexity, the direct effect of education on dementia was almost fully attenuated in both logistic and mediation models. Health outcomes, health behaviours and income also mediated the association, explaining 29%, 35% and 10% respectively. Thus, we highlight four pathways through which a significant proportion of the impact of education on all-cause dementia can be explained.

These percentages add up to over 100% as the different mediators were considered independent of each other. For example, occupational complexity explains 74% of the association between education and dementia independent of health behaviours and health outcomes.

Contrary to what we expected, social isolation does not appear to mediate the relationship. The association between social isolation and education was very small, therefore social isolation has little mediatory effect.

### Sensitivity analysis

Results from sensitivity analysis on complete case data differ somewhat from the results of analysis on imputed data. Participants with complete case data have higher education and more complex occupations. Therefore, analysis using complete case data has less power to detect associations among those with lower occupational complexities and less education which could have resulted in different results to those obtained using imputed data. 95% confidence intervals for mediation analysis of complete case data encompassed point estimates from the analysis on imputed data for all mediators aside from income however, they were wide. Alternatively, unmeasured confounding could contribute to differences between the two analyses. Education and occupational complexity are both proxies for SES, indicating the SES of those with complete case data is higher than that for those with missing data. SES does not map perfectly onto education or income and so residual confounding by other factors such as wealth may exist.

### Current literature

In line with prior work, we find occupational complexity to mediate much of the association between education and dementia. A 2022 meta-analysis found occupational complexity to mediate 28% of the relationship between education and dementia [23] and an additional study estimated the proportion mediated to be between 11% and 22% [24]. We found occupational complexity to mediate a much higher proportion of the relationship, almost fully attenuating the association independent of this pathway. This could be driven by our larger sample size or by differences in sample characteristics. Previous work found the proportion mediated by occupational complexity was higher in white men [24]. Our study is 96% white, whereas prior studies were more diverse, therefore this may account for some of the difference [23, 24]. Amongst our largely white cohort, most of the relationship between education and dementia appears to be mediated by occupational complexity. However, alternate pathways may be more important for other ethnic groups.

Existing studies considering occupational complexity as a mediator do not adjust for health behaviours or health outcomes. We build on this work by considering health factors as confounders and observe an association independent of pathways involving in health. This aligns with prior work which found that modifiable health factors only partly explained the relationship between socioeconomic status and dementia [20]. Additionally, the mediatory effect of occupational complexity is far greater than that of income, suggesting it is the complexity of the role an individual is performing as opposed to the income obtained from their role that influences dementia risk. Together, these findings demonstrate a pathway from education to dementia through occupational complexity, independent of interplay with health or income. This can be explained through the cognitive reserve hypothesis. Those with greater occupational complexity have greater mental stimulation, increasing cognitive reserve and protecting against dementia [3]. It has been found that participating in cognitively stimulating activities in mid-life improved late-life cognitive ability independent of occupation and education level therefore, interventions based on cognitively stimulating activities should be targeted to those with less education and lower occupational complexity to counteract this pathway [45]. The magnitude of the mediatory effect of occupational complexity may also result from our dichotomisation of education. We compared those with education before and after the age of 16. Having education post 18 could strongly predict occupational complexity and so by using 16 as the cut-off, not 18, we could overestimate the indirect effect of education on dementia through occupational complexity.

Additional to a pathway through occupational complexity, we find evidence to support the role of income, health behaviour and outcomes as mediators. Few studies consider income as a mediator of the association between education and dementia. Income has been considered alongside education in proxy SES measures, but this does not factor in the temporal sequence of events [20, 21]. One study found an indirect pathway between education and dementia via wealth and LIBRA score. This suggests that those who have higher education have a higher income which leads to them adopting better health behaviours, reducing their risk of dementia [21]. Contrary to this, we found income to mediate 10% of the relationship independent of health outcomes and health behaviours. The association between income and dementia has been shown to persist upon adjustment for health-related risk factors and so alternate pathways likely exist [46]. Exposure to negative life events and poor environmental conditions in association with low income may provoke chronic stress responses resulting in physiological dysregulation and cognitive decline [47]. However, it is most likely that associations with income are driven by occupational complexity. To avoid collinearity, occupation complexity was not included as a confounder in the income mediation model. Thus, given the large mediatory effect of occupational complexity, it is likely that it also drives the mediatory effect of income. Additionally, income is not a perfect marker of SES and given a large proportion of our sample are retired, wealth may not be reflected in this metric. This would also reduce the proportion of the association mediated through income.

Health-related lifestyle factors have previously been found to mediate the association between education and dementia to a small extent [16, 17, 20, 21]. However, this work often uses collated metrics of health outcomes and behaviours, therefore the respective effects of each could not be established [16, 18, 20, 21, 48]. We find that health outcomes and health behaviours both mediate the association between education and dementia independent of each other. We considered all health conditions that were present on the list of potentially modifiable risk factors for dementia modelled on the 2020 Lancet Commission on dementia prevention, intervention, and care [33]. Given the association between education and many of these conditions, we expected these health conditions to mediate the association between education and dementia risk [49–55]. However, our work also suggests that health behaviours impact dementia risk in association with education independent of these conditions. Aligning with this, one study found cardiovascular risk factors had a larger mediatory effect than cardiovascular health event [16]. Health behaviours could be acting to increase risk of dementia directly or via health conditions not considered in this study.

Future research should further unpick the examined associations. Investigation into the pathway through occupational complexity should be further examined to determine for whom this pathway is most prevalent. The relative importance of each potential mediator should be determined for different ethnicities, enabling more targeted and effective intervention. In addition, studies looking into whether health behaviours are protective in themselves or if they mediate the relationship via health conditions not considered in this study should be carried out.

### Strengths and limitations

The large sample size from UKB, maximised by use of multiple imputation, could improve the reliability and precision of our findings. Additionally, we used electronic records to improve confidence in health outcomes reporting. Data on education and each of the mediators was obtained prior to dementia diagnosis, clarifying the temporal sequence of events. Additionally, although education was reported at baseline, it would have occurred prior to enrolment in the study and development of mediators and confounders.

Despite the strengths of this study, limitations are present. Many variables, including education, were measured through self-report, introducing recall bias. The prodromal phase of dementia can consist of almost 20 years of pre-clinical neuropathology development. Therefore, our exclusion criteria of one year may not have been sufficient and the effects observed in this study may be stronger in reality. It is possible that individuals may have taken a simpler, lower paying job as their memory started to decline resulting in reverse causality. Alternatively, participants could retire or transition to reduced hours meaning our study would still consider their most complex, highest paying role. Additionally, our measure of occupational complexity reflects occupational attainment, not the intellectual requirements of the job. Future studies which build on our work should consider the intellectual requirements of occupations.

Cohort effects could be present as the operationalisation of education differentially selects younger individuals into the higher education group. When UKB recruited volunteers the response rate was only 5.5% [56]. Individuals who responded are likely to be more educated and to have better health compared to those who did not respond and consequently our findings may not be representative of the UK population. However, many findings from UKB appear to be generalisable to England and Scotland [57]. Additionally, within our mediation models, we mutually adjust for mediators. This methodological choice could result in collider bias and may induce non causal associations. However, not mutually adjusting for mediators would have resulted in unmeasured confounding, violating the other assumptions of causal mediation analysis. Additional studies should be conducted using alternative data sources to observer whether our findings can be replicated.

## Conclusions

The pathway from education to all-cause dementia is complex and multifactorial. The results of this study highlight multiple pathways through which an individual’s risk of dementia could be reduced. In alignment with the cognitive reserve hypothesis, much of the association is mediated by occupational complexity. The mental stimulation obtained through complex work appears increase an individual’s cognitive reserve, increasing their resilience to the neuropathological changes associated with dementia. Therefore, intervention to increase access to more complex work or mentally stimulating leisure activities amongst those with lower levels of education could prove beneficial [44]. Acting to increase the cognitive reserve of those with less education, particularly those working in a low complexity job, could reduce the number of people in the UK living with dementia. Additionally, increasing access to education and intervening to improve the health of those with less education may reduce dementia risk in those most vulnerable. This is of vital importance given the increasing pressure dementia places on the NHS, social care system, unpaid carers and those living with the condition.

## Electronic supplementary material

Below is the link to the electronic supplementary material.

Supplementary Material 1

Supplementary Material 2

## Data Availability

Due to the sensitive and personal nature of this data, it is not publicly available. However, data from UK Biobank (http://www.ukbiobank.ac.uk/) are available to all researchers upon application. This research was carried out under Application Number 40055.

## Abbreviations

SES: Socioeconomic status
LIBRA: Poly-environmental risk score
UKB: UK biobank
SOC: Standard occupational code
DAG: Directed acyclic graphs
ACME: Average causal mediatory effect
ADE: Average direct effect
