# Supplementary material for: Mediation of the association between education and dementia by occupational complexity, income, health behaviours and health outcomes
Source: BMC Psychiatry. 2025 Feb 25;25:174. doi: 10.1186/s12888-025-06619-4 (PMC11863402; doi:10.1186/s12888-025-06619-4)
Supplement: Supplementary file 1 — Supplementary Material 1 [file 12888_2025_6619_MOESM1_ESM.docx]

**Additional File 1**

**Additional Tables**

|  | **Complete Case Data** | | **Imputed Data** |
| --- | --- | --- | --- |
| **Variables** | **N (Proportion of sample)** | **Missing values (%)** | **Proportion of sample** |
| **Dementia** |  |  |  |
| No dementia | 22302 (1.0%) | 0 (0.0%) | 2.0% |
| Dementia | 221047(99.0%) |  | 98.0% |
| **Education** |  |  |  |
| Education after 16 | 137172(61.4%) | 2583 (0.7%) | 54.3% |
| Education up 16 | 86177(38.6%) |  | 45.7% |
| **Sex** |  |  |  |
| Female | 114280 (51.2%) | 0 (0.0%) | 54.3% |
| Male | 109069 (48.8%) |  | 45.7% |
| **Age** | 58.5 (5.2) | 0 (0.0%) | 60.0 (0.01) |
| Mean (SE) |  |  |  |
| **Ethnicity** |  |  |  |
| Caucasian | 215158 (96.3%) | 2595 (0.7%) | 96.0% |
| Not Caucasian | 8191 (3.7%) |  | 4.0% |
| **Income** |  |  |  |
| <31,000 | 101518 (45.5%) | 60107 (15.6%) | 45.3% |
| >30,999 | 121831 (54.5%) |  | 54.6% |
| **Occupational Complexity** |  |  |  |
| Managerial & professional | 128320 (57.5%) | 114724 (29.9%) | 37.7% |
| Non managerial or professional occupations | 95029 (42.5%) |  | 62.3% |
| **Social Isolation** |  |  |  |
| Not socially isolated | 186293(83.4%) | 753(0.2%) | 83.4% |
| Socially isolated | 37056 (16.6%) |  | 16.6% |
| **Health Outcomes** |  |  |  |
| Good/Excellent Health | 192825 (86.3%) | 0 (0.0%) | 84.7% |
| Intermediate/Poor health | 30524 (13.7%) |  | 15.3% |
| **Health Behaviour** |  |  |  |
| Favourable | 191536 (85.8%) | 23987 (6.2%) | 80.8% |
| Unfavourable | 31813 (14.2%) |  | 19.2% |
| **Depression** |  |  |  |
| No | 204991 (91.8%) | 0 (0.0%) | 93.7% |
| Yes | 18358 (8.2%) |  | 6.3% |

*Supplementary table 1. Sample characteristics of complete case and imputed data*

| **Model** | **OR** | **95% CI** | |
| --- | --- | --- | --- |
| Adjusted for Age, Sex & Ethnicity | 0.69 | 0.64, | 0.75 |
| + Health Outcome | 0.71 | 0.66, | 0.78 |
| + Health Outcome & Social Isolation | 0.71 | 0.66, | 0.78 |
| + Health Outcome, Social Isolation & Health Behaviour | 0.72 | 0.66, | 0.79 |
| + Health Outcome, Social Isolation, Health Behaviour & Income | 0.81 | 0.74, | 0.89 |
| + Health Outcome, Social Isolation, Health Behaviour & Occupation | 0.86 | 0.78, | 0.94 |

*Supplementary table 2. Logistic regression of dementia in association with education on complete case data*

|  | **β** | **95% CI** | | **Proportion Mediated (95% CI)** |
| --- | --- | --- | --- | --- |
| ***Occupational Complexity as a mediator*** |  |  |  |  |
| Total Effect | 0.0034 | 0.0025 | 0.0043 | 54% (39%-76%) |
| Average Direct Effect | 0.0016 | 0.0007 | 0.0026 |  |
| Average Causal Mediation Effect | 0.0018 | 0.0014 | 0.0022 |  |
| ***Income as a mediator*** |  |  |  |  |
| Total Effects | 0.0034 | 0.0025 | 0.0042 | 39% (29%-54%) |
| Average Direct Effect | 0.0013 | 0.0011 | 0.0015 |  |
| Average Causal Mediation Effect | 0.0021 | 0.0012 | 0.0029 |  |
| ***Health Outcomes as a mediator*** |  |  |  |  |
| Total Effects | 0.0012 | 0.0003 | 0.0021 | 25% (13%-120%) |
| Average Direct Effect | 0.0009 | -0.0001 | 0.0017 |  |
| Average Causal Mediation Effect | 0.0003 | 0.0002 | 0.0004 |  |
| ***Social Isolation as a mediator*** |  |  |  |  |
| Total Effects | 0.0039 | 0.0030 | 0.0047 | 1% (0%-1%) |
| Average Direct Effect | 0.0039 | 0.0030 | 0.0047 |  |
| Average Causal Mediation Effect | 0.0000 | 0.0000 | 0.0000 |  |
| ***Health Behaviours as a mediator*** |  |  |  |  |
| Total Effects | 0.0010 | 0.0001 | 0.0020 | 12% (1%-73%) |
| Direct Effects | 0.0009 | 0.0000 | 0.0019 |  |
| Average Causal Mediation Effect | 0.0001 | 0.0000 | 0.0002 |  |

*Supplementary table 3. Mediation analysis on complete case data.*

| **Significance test for mediator-outcome interaction** | **Imputed data** | | | | **Complete case data** | | | |
| --- | --- | --- | --- | --- | --- | --- | --- | --- |
|  | **Test statistic** | **95% CI** | | **Proportion mediated** | **Test statistic** | **95% CI** | | **Proportion mediated** |
| Health outcomes | -0.00007 | -0.00023 | 0.00009 | 27% | 0.00005 | -0.00010 | 0.00021 | 26% |
| Occupational complexity | -0.00058 | -0.00116 | -0.00001 | 72% | 0.00057 | -0.00141 | 0.00021 | 53% |
| Income | -0.00061 | -0.00094 | -0.00028 | 10% | -0.00057 | 0.00011 | 0.00104 | 39% |
| Health behaviours | -0.00009 | -0.00033 | 0.00015 | 36% | 0.00015 | -0.00003 | 0.00035 | 12% |
| Social isolation | 0.00001 | 0.00000 | 0.00002 | 0% | 0.00001 | 0.00000 | 0.00004 | 1% |

*Supplementary table 4. Mediation analysis testing for mediator-outcome interactions.*

**Additional Figures**

*
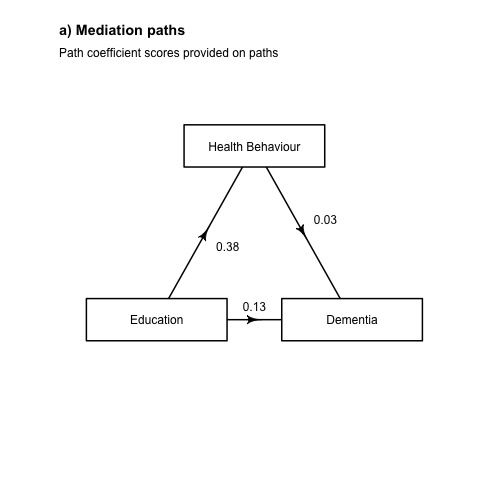

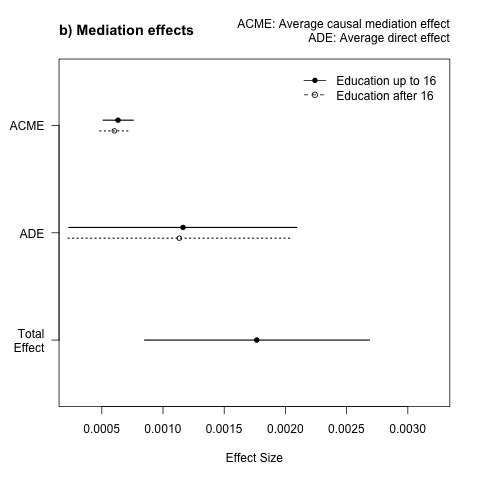
*

*Supplementary figure 1.a) Mediation paths of health behaviour as a mediator. b) Average causal mediation effect (ACME), average direct effect (ADE) for those with education up to and post the age of 16 and the total effect of education and health behaviours on dementia.*

*
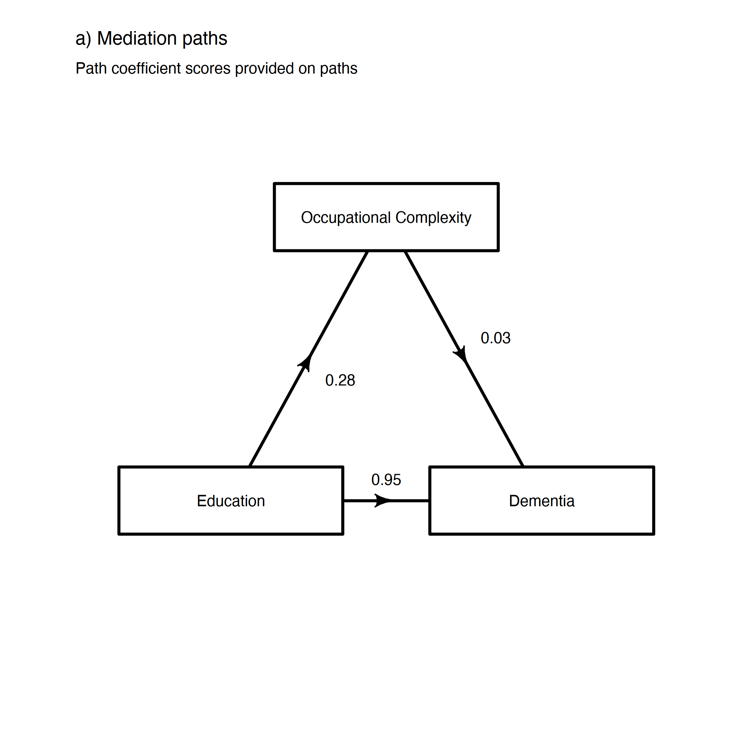

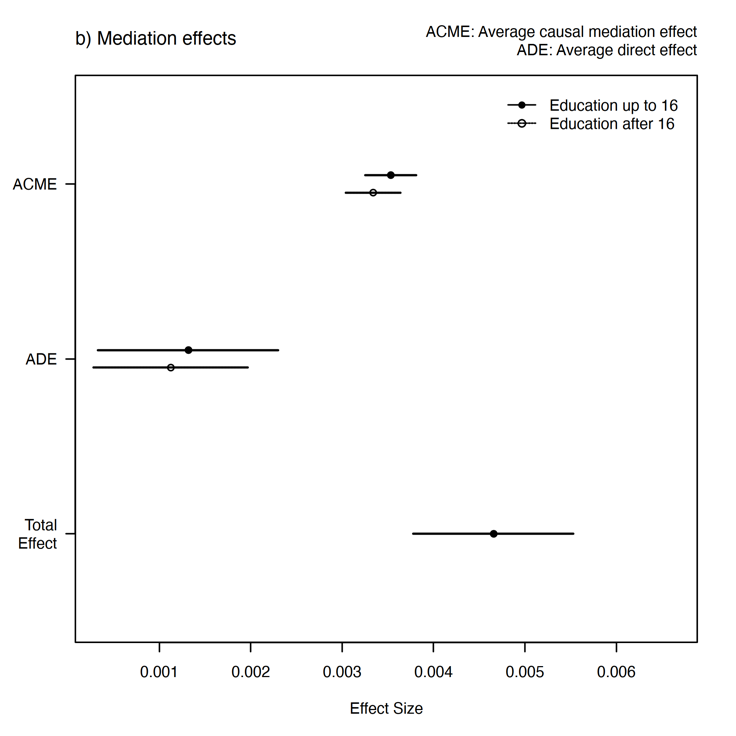
*

*Supplementary figure 2.a) Mediation paths of health outcome as a mediator. b) Average causal mediation effect (ACME), average direct effect (ADE) for those with education up to and post the age of 16 and the total effect of education and health outcomes on dementia.*

*
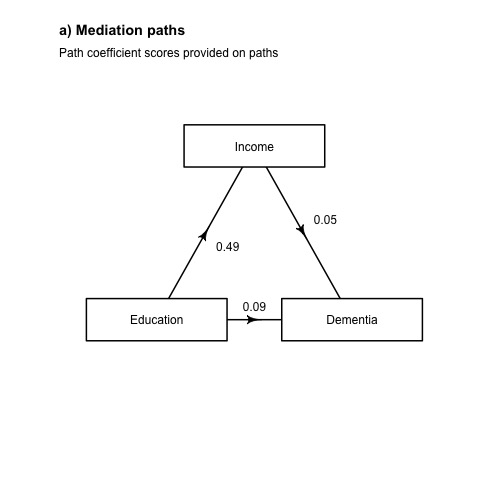

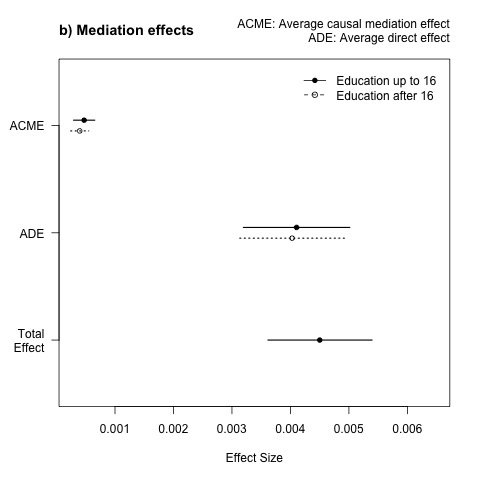
*

*Supplementary figure 3.a) Mediation paths of income as a mediator. b) Average causal mediation effect (ACME), average direct effect (ADE) for those with education up to and post the age of 16 and the total effect of education and income on dementia.*
