# Supplementary material for: Mediation of the association between education and dementia by occupational complexity, income, health behaviours and health outcomes
Source: BMC Psychiatry. 2025 Feb 25;25:174. doi: 10.1186/s12888-025-06619-4 (PMC11863402; doi:10.1186/s12888-025-06619-4)
Supplement: Supplementary file 2 — Supplementary Material 2 [file 12888_2025_6619_MOESM2_ESM.pdf]

```
# Dataset Compliation - (Code 1) ####################################################################

## --------- Set up ---------------------------------------------------------------------------------

library(haven)
library(dplyr)
library(dtplyr)
library(finalfit)
library(summarytools)
library(matrixStats)
library(hablar)
library(naniar)

#Set working directory
setwd("/Volumes/GroupFolders/MHS_Naaheed_UKB/Katherine Taylor")
getwd()

## --------- Data loading and refinement   ------------------------------------------------------

#Variables included - select only variables required.

## Health behaviors
health_behaviours_q <-
c("n_1309_0_0","n_1319_0_0","n_1329_0_0","n_1339_0_0","n_1349_0_0","n_1389_0_0","n_1289_0_0","n_1299_0_0","n_1448_0_0","n_1438_0_0","n

## Health outcomes

health_outcome_q <- c("obese","dm","htn","depression")

## Wealth
wealth_q <- c("townsend","town_quint","avg_household_income","n_738_1_0","n_738_2_0","n_10877_0_0")

## Occupational complexity
no_occ_soc_q <- seq(0,39)

for (number in no_occ_soc_q) {
  occupation_qa <- gsub(" ", "",paste('n_22617_0_',no_occ_soc_q))
}

current_occ_status <- c("n_6142_0_0","n_6142_0_1","n_6142_0_2","n_6142_0_3","n_6142_0_4","n_6142_0_5","n_6142_0_6")

occupation_q <- c("baseline_jobcode","n_20119_0_0",current_occ_status,occupation_qa)

#Combine baseline job code, historical job code, current employment status

## Social Isolation
isolation_q <- c("social","soc_isolation")

## Education
education_q <- c("edu_cat","edu_cat1","education")

## Dementia

dementia_q <-c("alldem","startdate","date_dementia")

## Covaraites

covariate <- c("sex","age","ageband","apoe","ethnicity")

## Exclusion criteria

exclusion <- "_merge"

#Load data
setwd("/Volumes/GroupFolders/MHS_Naaheed_UKB/Katherine Taylor/Data")
raw_data_1 <- read_stata("all_vars_merged_jan23.dta",col_select=c(id,all_of(health_behaviours_q),all_of(health_outcome_q),
all_of(wealth_q), all_of(occupation_q),all_of(isolation_q), all_of(education_q), all_of(dementia_q), all_of(covariate),
all_of(exclusion)))

#Exclude those who dropped out
raw_data <- raw_data_1 %>%
  filter(`_merge` != 1)

## --------- Variable cleaning   ------------------------------------------------------

### ========= Education data ============================================================

## Take the smallest number as that is the highest level of education, excluding -7 and -3.


Education <- raw_data %>%
  select(id,all_of(education_q)) # %>%
# na.omit()

## Summarise
Education_sum <- Education %>%
  group_by(edu_cat1) %>%
  summarise(count = n(),
            .groups='drop') %>%
  mutate(freq = count / sum(count))

## Collate categories into 3
Education2 <- Education %>%
  mutate(edu_cat2 = case_when(edu_cat1 %in% c(1,2,3) ~ 0,
                              edu_cat1 == 4 ~ 1,
                              edu_cat1 == 5 ~ 2))

### edu_cat 2 key: 0 - GSCE education or lower, 1 - completed secondary education, 2 - higher education.

## Summarize again
Education_sum2 <- Education2 %>%
  group_by(edu_cat2) %>%
  summarise(count = n(),
```

```
          .groups='drop') %>%
  mutate(freq = count / sum(count))

## Keep only those without missing education data
#edu_data_available <- Education %>%
#select(id)

### ========= Dementia data ==========================================================================

Dementia <- raw_data %>%
  select(id,alldem) %>%
  # filter(id %in% edu_data_available$id) %>%
  na.omit()

Recentdem <- raw_data %>%
  select(id,alldem,startdate,date_dementia) %>%
  # filter(id %in% edu_data_available$id) %>%
  na.omit() %>%
  mutate(date_dementia_td = as.Date(date_dementia, origin = "1960-01-01"),
         date_diff = date_dementia_td - startdate,
         exclude = case_when(date_diff<366 ~1,
                             date_diff >365 ~ 0))

Dementia_excl <- Recentdem %>%
  group_by(exclude) %>%
  summarise(count = n()) %>%
  mutate(freq = count / sum(count))

id_to_exclude <- Recentdem %>%
  filter(exclude == 1) %>%
  select(id)

## Keep only those without missing education and dementia data
edu_dem_data_available <- Dementia %>%
  select(id) %>%
  filter(!(id %in% id_to_exclude$id))

## Summarise
Dementia_sum <- Dementia %>%
  group_by(alldem) %>%
  summarise(count = n()) %>%
  mutate(freq = count / sum(count))

### dementia key: 1 = dementia, 0 = no dementia

### ========= Health Outcomes  ==========================================================================

# the higher the score, the worse the health outcome

## adu - available data used
## anyna_na - any missing data leads to coded as missing


Health_outcomes <- raw_data %>%
  select(id,c(any_of(health_outcome_q))) %>%
  filter(id %in% edu_dem_data_available$id)%>%
  # na.omit() %>%
  mutate(depression2 = case_when(depression != 0 ~ 1,
                                 depression == 0 ~ 0),
         ho_summary_adu = rowSums(.[2:4],6),
         ho_summary_anyna_na = rowSums(.[2:4], na.rm=FALSE))

missingd <- Health_outcomes %>%
  filter(is.na(depression))

## Keep only those without missing education and dementia data
#ho_data_available <- Health_outcomes %>%
#select(id)

## Summarise
Health_outcomes_sum <- Health_outcomes %>%
  group_by(ho_summary_anyna_na, ho_summary_adu ) %>%
  summarise(count = n(),
          .groups='drop') %>%
  mutate(freq = count / sum(count))


### high score - worse health

### ================ Health Behaviours ==========================================================================

Health_behaviours <- raw_data %>%
  select(id,c(any_of(health_behaviours_q))) %>%
  filter(id %in% edu_dem_data_available$id)

## Diet Cleaning -> 1 = good, 0 == bad

### Fruit - 3 or more portions

#### Where there is one only one variable of the two available, keep in the dataset
#### and assume they don't have any of this food.

### Include comparison where any missing data leads to na

Fruit <- Health_behaviours %>%
  mutate(freshfruit = case_when(n_1309_0_0 == -10 ~ 0,
                                n_1309_0_0 %in% c(-3,-1) ~ as.double(NA),
                                TRUE ~ n_1309_0_0 ),
         driedfruit = case_when(n_1319_0_0 == -10 ~ 0,
                                n_1319_0_0 %in% c(-3,-1) ~ as.double(NA),
                                TRUE ~ n_1319_0_0)) %>%
  mutate(fruit_adu = case_when(!if_all(c(freshfruit, driedfruit),is.na) ~ rowSums(.[19:20],6)),
         fruit_anyna_na = rowSums(.[19:20], na.rm=FALSE),
         fruit_cat_adu = case_when(fruit_adu > 2 ~ 1,
```

```
                                    fruit_adu <3 ~ 0 ),
         fruit_cat_anyna_na = case_when(fruit_anyna_na > 2 ~ 1,
                                         fruit_anyna_na <3 ~ 0 ))  %>%
  select(id,n_1309_0_0,n_1319_0_0,freshfruit,driedfruit,fruit_adu,fruit_anyna_na,fruit_cat_adu,fruit_cat_anyna_na)


#### Summarise
Fruit_sum <- Fruit %>%
  group_by(fruit_cat_adu,fruit_cat_anyna_na) %>%
  summarise(count = n(),
            .groups='drop') %>%
  mutate(freq = count / sum(count))


### Vegetables - 3 or more portions

Veg <- Health_behaviours %>%
  mutate(cookedveg = case_when(n_1289_0_0 == -10 ~ 0,
                               n_1289_0_0 %in% c(-3,-1) ~ as.double(NA),
                               TRUE ~ n_1289_0_0 ),
         rawveg = case_when(n_1299_0_0 == -10 ~ 0,
                            n_1299_0_0 %in% c(-3,-1) ~ as.double(NA),
                            TRUE ~ n_1299_0_0 )) %>%
  mutate(veg_adu = case_when(!if_all(c(cookedveg, rawveg),is.na) ~ rowSums(.[19:20],6)),
         veg_anyna_na = rowSums(.[19:20], na.rm=FALSE),
         veg_cat_adu = case_when(veg_adu > 2 ~ 1,
                                 veg_adu <3 ~ 0 ),
         veg_cat_anyna_na = case_when(veg_anyna_na > 2 ~ 1,
                                      veg_anyna_na <3 ~ 0 )) %>%
  select(id,n_1289_0_0,n_1299_0_0,cookedveg,rawveg,veg_adu,veg_anyna_na,veg_cat_adu,veg_cat_anyna_na)


#### Summarise
Veg_sum <- Veg %>%
  group_by(veg_cat_adu,veg_cat_anyna_na) %>%
  summarise(count = n(),
            .groups='drop') %>%
  mutate(freq = count / sum(count))


### Fish - 2 or more portions

Fish <- Health_behaviours %>%
  #head(1000) %>%
  select(id,n_1339_0_0,n_1329_0_0) %>%
  mutate(nonoilfish = case_when(n_1339_0_0 %in% c(-3,-1) ~ as.double(NA),
                                TRUE ~ n_1339_0_0 ),
         oilfish = case_when(n_1329_0_0 %in% c(-3,-1) ~ as.double(NA),
                             TRUE ~ n_1329_0_0),
         fishcat_anyna_na = case_when(is.na(nonoilfish) ~ as.double(NA),
                                      is.na(oilfish) ~ as.double(NA),
                                      nonoilfish >2 | oilfish > 2 ~ 1,
                                      oilfish == 2 & nonoilfish == 2 ~ 1,
                                      TRUE ~ 0),
         fishcat_cat_adu = case_when(is.na(nonoilfish) & is.na(oilfish) ~ as.double(NA),
                                     nonoilfish >2 | oilfish > 2 ~ 1,
                                     oilfish == 2 & nonoilfish == 2 ~ 1,
                                     TRUE ~ 0))

#### Summarise
Fish_sum <- Fish %>%
  group_by(fishcat_anyna_na,fishcat_cat_adu) %>%
  summarise(count = n(),
            .groups='drop') %>%
  mutate(freq = count / sum(count))

### Processed meat - less than once a week

Procmeat <- Health_behaviours %>%
  #head(1000) %>%
  select(id,n_1349_0_0) %>%
  mutate(procmeat = case_when(n_1349_0_0 %in% c(-3,-1) ~ as.double(NA),
                              TRUE ~ n_1349_0_0 )) %>%
  mutate(procmeat_cat = case_when(is.na(procmeat) ~ as.double(NA),
                                  procmeat >2 ~ 0,
                                  TRUE ~ 1))

#### Summarise
Procmeat_sum <- Procmeat %>%
  group_by(procmeat_cat) %>%
  summarise(count = n(),
            .groups='drop') %>%
  mutate(freq = count / sum(count))

### Unprocessed red meat - less than 1.5 times a week

Redmeat <- Health_behaviours %>%
  #head(1000) %>%
  select(id, n_1369_0_0,n_1379_0_0,n_1389_0_0) %>%
  mutate(beef = case_when(n_1369_0_0  %in% c(-3,-1) ~ as.double(NA),
                          TRUE ~ n_1369_0_0),
         lamb = case_when(n_1379_0_0    %in% c(-3,-1) ~ as.double(NA),
                          TRUE ~ n_1379_0_0),
         pork = case_when(n_1389_0_0 %in% c(-3,-1) ~ as.double(NA),
                          TRUE ~ n_1389_0_0)) %>%
  mutate(redmeat_cat_anyna_na = case_when(is.na(beef) ~ as.double(NA),
                                          is.na(lamb) ~ as.double(NA),
                                          is.na(pork) ~ as.double(NA),
                                          beef > 2 | lamb > 2 | pork > 2 ~ 0,
                                          beef==2 & lamb == 2 ~ 0,
                                          beef ==2 & pork == 2 ~ 0,
                                          lamb == 2 & pork == 2 ~ 0,
                                          TRUE ~ 1),
         redmeat_cat_adu = case_when(is.na(beef) & is.na(lamb) & is.na(pork) ~ as.double(NA),
```

```
                                        beef > 2 | lamb > 2 | pork > 2 ~ 0,
                                        beef==2 & lamb == 2 ~ 0,
                                        beef ==2 & pork == 2 ~ 0,
                                        lamb == 2 & pork == 2 ~ 0,
                                        TRUE ~ 1))

#### Summarise
redmeat_sum <- Redmeat %>%
  group_by(redmeat_cat_anyna_na,redmeat_cat_adu) %>%
  summarise(count = n(),
            .groups='drop') %>%
  mutate(freq = count / sum(count))


### Whole grains - three or more savings per day
### Refined grains - less than 1.5 servings per day

Grains <- Health_behaviours %>%
  #head(1000) %>%
  select(id,n_1438_0_0,n_1458_0_0,n_1448_0_0,n_1468_0_0) %>%
  mutate(breadintake = case_when(n_1438_0_0 == -10 ~ 0,
                                 n_1438_0_0  %in% c(-3,-1) ~ as.double(NA),
                                 TRUE ~ n_1438_0_0),
         cerealintake = case_when( n_1458_0_0  == -10 ~ 0,
                                   n_1458_0_0   %in% c(-3,-1) ~ as.double(NA),
                                   TRUE ~  n_1458_0_0 ),
         wholegrainbread = case_when(is.na(n_1448_0_0) ~ as.double(NA),
                                     n_1448_0_0 %in% c(2,3) ~ 1,
                                     TRUE ~ 0),
         refinedbread = case_when(is.na(n_1448_0_0) ~ as.double(NA),
                                  n_1448_0_0 == 1 ~ 1,
                                  TRUE ~ 0),
         wholegraincereal = case_when(is.na(n_1468_0_0) ~ as.double(NA),
                                      n_1468_0_0 %in% c(1,3,4) ~ 1,
                                      TRUE ~ 0),
         refinedcereal = case_when(is.na(n_1468_0_0) ~ as.double(NA),
                                   n_1468_0_0 %in% c(2,5) ~ 1,
                                   TRUE ~ 0)) %>%
  mutate(totalgrain_anyna_na = rowSums(.[6:7], na.rm=FALSE),
         totalgran_adu = rowSums(.[6:7], na.rm=TRUE),
         wholegrain_cat_anyna_na = case_when(is.na(totalgrain_anyna_na) ~ as.double(NA),
                                             wholegrainbread==1 & breadintake >2 |
                                               wholegraincereal==1 & cerealintake >2|
                                               wholegrainbread == 1 & wholegrainbread == 1 & totalgrain_anyna_na > 2 ~ 1,
                                             TRUE ~ 0),
         wholegrain_cat_adu = case_when(is.na(breadintake) & is.na(cerealintake) ~ as.double(NA),
                                        wholegrainbread==1 & breadintake >2 |
                                          wholegraincereal==1 & cerealintake >2|
                                          wholegrainbread == 1 & wholegrainbread == 1 & totalgran_adu > 2 ~ 1,
                                        TRUE ~ 0),
         refined_cat_anyna_na = case_when(is.na(totalgrain_anyna_na) ~ as.double(NA),
                                          refinedbread==1 & breadintake > 2 |
                                            refinedcereal==1 &  cerealintake > 2 |
                                            refinedbread==1 & refinedcereal==1 & totalgrain_anyna_na > 2 ~ 0,
                                          TRUE ~ 1),
         refined_cat_adu = case_when(is.na(breadintake) & is.na(cerealintake) ~ as.double(NA),
                                     refinedbread==1 & breadintake > 2 |
                                       refinedcereal==1 &  cerealintake > 2 |
                                       refinedbread==1 & refinedcereal==1 & totalgran_adu > 2 ~ 0,
                                     TRUE ~ 1))



#### Summarise
wholegrain_sum <- Grains %>%
  group_by(wholegrain_cat_anyna_na,wholegrain_cat_adu) %>%
  summarise(count = n(),
            .groups='drop') %>%
  mutate(freq = count / sum(count))

refined_sum <- Grains %>%
  group_by(refined_cat_anyna_na,refined_cat_adu) %>%
  summarise(count = n(),
            .groups='drop') %>%
  mutate(freq = count / sum(count))

### Join together diet data

Diet <- edu_dem_data_available %>% left_join(Fruit,
                                             by='id') %>% left_join(Veg,
                                                                    by='id') %>% left_join(Fish,
                                                                                           by = 'id') %>% left_join(Procmeat,
                                                                                                                    by = 'id') %>%
left_join(Redmeat,

by = 'id') %>% left_join(Grains,

by = 'id') #%>%
#select(id,fruit_cat,veg_cat,fish_cat,procmeat_cat,redmeat_cat,wholegrain_cat,refined_cat)

###Examining missing data
explanatory = c("fruit_cat_adu","fruit_cat_anyna_na","veg_cat_adu","veg_cat_anyna_na",
                "fishcat_anyna_na","fishcat_cat_adu","procmeat_cat",
                "redmeat_cat_anyna_na","redmeat_cat_adu", "wholegrain_cat_anyna_na","wholegrain_cat_adu",
                "refined_cat_anyna_na","refined_cat_adu")
dependent = "id"

Diet %>%
  ff_glimpse(dependent, explanatory)

### Less than 1% missing for all varaibles - remove those with missing data.


### Create summary diet variable
```

```
Diet2 <- Diet %>%
  #na.omit() %>%
  mutate(diet_score_adu = rowSums(.[,c(8,16,23,26,34,48,50)], na.rm=TRUE),
         diet_adu = case_when(is.na(diet_score_adu) ~ as.double(NA),
                              diet_score_adu<4 ~ 1,
                              TRUE ~ 0),
         diet_score_anyna_na = rowSums(.[,c(9,17,22,26,33,47,49)], na.rm = FALSE),
         diet_anyna_na = case_when(is.na(diet_score_anyna_na) ~ as.double(NA),
                                   diet_score_anyna_na<4 ~ 1,
                                   TRUE ~ 0))

test <- Diet2 %>%
  filter(diet_score_anyna_na != diet_score_adu)

Diet_sum <- Diet2 %>%
  group_by(diet_score_adu,diet_score_anyna_na) %>%
  summarise(count = n(),
            .groups='drop') %>%
  mutate(freq = count / sum(count))

Diet_sum2 <- Diet2 %>%
  group_by(diet_adu,diet_anyna_na) %>%
  summarise(count = n(),
            .groups='drop') %>%
  mutate(freq = count / sum(count))

# No cases with no data
check <- Diet2[rowSums(is.na(Diet2)) != ncol(Diet2), ]

### Create summary health behaviour variable - the higher the number, the worse the health behaviour
Health_behaviours2 <- Diet2 %>% left_join(Health_behaviours,
                                          by ='id') %>%
  select(id,currsmok,inactivity,alcohol,diet_adu,diet_anyna_na)

#### Assess missing data situation

explanatory2 <- c("currsmok","inactivity","alcohol","diet_adu","diet_anyna_na")

Health_behaviours2 %>%
  ff_glimpse(dependent, explanatory2)

##### Only smoking has missing data (0.3%)

Health_behaviours3 <- Health_behaviours2%>%
  #  na.omit()%>%
  mutate(healthbehaviour_anyna_na = rowSums(.[,c(2,3,4,6)],na.rm = FALSE),
         healthbehaviour_adu = rowSums(.[,c(2,3,4,5)],na.rm = TRUE),
         healthbehaviourcat_anyna_na = case_when(is.na(healthbehaviour_anyna_na)~ as.double(NA),
                                                 healthbehaviour_anyna_na== 0 | healthbehaviour_anyna_na== 1 ~ 0,
                                                 healthbehaviour_anyna_na== 2 ~ 1,
                                                 healthbehaviour_anyna_na== 3 |healthbehaviour_anyna_na== 4 ~ 2),
         healthbehaviourcat_adu = case_when(is.na(healthbehaviour_adu)~ as.double(NA),
                                            healthbehaviour_adu== 0 | healthbehaviour_adu== 1 ~ 0,
                                            healthbehaviour_adu== 2 ~ 1,
                                            healthbehaviour_adu== 3 |healthbehaviour_adu== 4 ~ 2))




#### Summarise - 0 = favourable, 1 = Intermediate, 2 = Unfavourable
Health_behaviour_sum <- Health_behaviours3 %>%
  group_by(healthbehaviourcat_adu,healthbehaviourcat_anyna_na) %>%
  summarise(count = n(),
            .groups='drop') %>%
  mutate(freq = count / sum(count))


#hb_data_available <- Health_behaviours3 %>%
#select(id)

### =========================== Social Isolation =====================================================

Isolation <- raw_data %>%
  select(id,social) %>%
  filter(id %in% edu_dem_data_available$id) #%>%
#na.omit()

Isolation_sum <- Isolation %>%
  group_by(social) %>%
  summarise(count = n(),
            .groups='drop') %>%
  mutate(freq = count / sum(count))

## 1 = socially isolated

#si_data_available <- Isolation %>%
#  select(id)

### =========================== Wealth =================================================================

Wealth <- raw_data %>%
  select(id,c(any_of(wealth_q))) %>%
  filter(id %in% edu_dem_data_available$id) %>%
  mutate(income_1 = case_when(avg_household_income %in% c(-3,-1) ~ as.double(NA),
                              TRUE ~ avg_household_income),
         income_4 = case_when(n_10877_0_0 %in% c(-3,-1) ~ as.double(NA),
                              TRUE ~ n_10877_0_0),
         income_2 = case_when(n_738_1_0 %in% c(-3,-1) ~ as.double(NA),
                              TRUE ~ n_738_1_0),
         income_3 = case_when(n_738_2_0 %in% c(-3,-1) ~ as.double(NA),
                              TRUE ~ n_738_2_0),
         avg_household_income_c1 = case_when(is.na(income_1) ~ 0,
                                             TRUE ~ income_1),
```

```
         avg_household_income_c2 = case_when(avg_household_income_c1==0 ~ income_2,
                                             TRUE ~ avg_household_income_c1),
         avg_household_income_c3 = case_when(is.na(avg_household_income_c2) ~ 0,
                                             TRUE ~ avg_household_income_c2),
         avg_household_income_c4 = case_when(avg_household_income_c3==0 ~ income_3,
                                             TRUE ~ avg_household_income_c3),
         avg_household_income_c5 = case_when(is.na(avg_household_income_c4) ~ 0,
                                             TRUE ~ avg_household_income_c4),
         avg_household_income_c = case_when(avg_household_income_c5==0 ~ income_4,
                                            TRUE ~ avg_household_income_c5))


Wealth_sum2 <- Wealth %>%
  group_by(income_1) %>%
  summarise(count = n(),
            .groups='drop') %>%
  mutate(freq = count / sum(count))

Wealth_sum <- Wealth %>%
  group_by(avg_household_income_c) %>%
  summarise(count = n(),
            .groups='drop') %>%
  mutate(freq = count / sum(count))

### Higher number = wealthier, should swap this around and refine categories (done in later code)

###  =========================== Occupational Complexity =====================================================

Occupation <- raw_data %>%
  select(id,c(any_of(occupation_q))) %>%
  filter(id %in% edu_dem_data_available$id)

Occupation2 <- Occupation %>%
  mutate(m_complex_hj = pmin(!!!select(., -c(1:10)), na.rm = TRUE))

Occupation3 <- Occupation2 %>%
  mutate(job_code = case_when(n_6142_0_0==1 ~ baseline_jobcode,
                              TRUE ~ m_complex_hj))

Occupation4 <- Occupation3 %>%
  select(c(1,2,51,52))

test <- Occupation4 %>%
  filter(m_complex_hj  == 0)

# Look at those still missing data

missing <- Occupation3 %>%
  filter(is.na(job_code)) %>%
  mutate(jobcode_r = case_when(!(is.na(baseline_jobcode)) ~ baseline_jobcode))

missing2 <- missing %>%
  filter(is.na(jobcode_r)|jobcode_r==0)

missing3 <- missing2 %>%
  replace_with_na(replace = list(n_6142_0_0 = c (-3,-7),n_6142_0_1 = c (-3,-7),
                                 n_6142_0_2 = c (-3,-7),n_6142_0_3 = c (-3,-7),
                                 n_6142_0_4 = c (-3,-7),n_6142_0_5 = c (-3,-7),
                                 n_6142_0_6 = c (-3,-7)))

missing4 <- missing3%>%
  mutate(min_stat = pmin(!!!select(., c(4:10)), na.rm = TRUE))

missing5 <- missing4 %>%
  select(c(1,2,3,4,5,6,7,8,9,10,54)) %>%
  mutate(unmployed_lag = case_when(min_stat>2 ~ 1,
                                   TRUE ~ 0))

Occupation5 <- Occupation4 %>%
  left_join(missing, by = "id") %>%
  select(c(1,4,56)) %>%
  left_join(missing5, by = "id") %>%
  select(c(1,2,3,13,14))

names(Occupation5)
colnames(Occupation5)[2] ="jobcode_a"

Occupation6 <- Occupation5 %>%
  mutate(jobcodef = case_when(!(is.na(jobcode_a)) ~ jobcode_a,
                              !(is.na(jobcode_r)) ~ jobcode_r))

Occupation7 <- Occupation6 %>%
  mutate (SOC_key = as.numeric(substr(jobcodef,1,1)))

Occupation8 <- Occupation7 %>%
  mutate(occupation_complexity = case_when(SOC_key %in% c(1,2,3) ~ 0,
                                           SOC_key %in% c(4,5,6) ~ 1,
                                           SOC_key %in% c(7,8,9) ~ 2,
                                           unmployed_lag == 1 ~ 3,
                                           TRUE ~ as.double(NA)))

### Missing data assessment

explanatory2 <- c("occupation_complexity")
dependent = "id"

Occupation8 %>%
  ff_glimpse(dependent, explanatory2)

Occupation9 <- Occupation8 %>%
  group_by(SOC_key) %>%
  summarise(count = n(),
            .groups='drop') %>%
  mutate(freq = count / sum(count))
```

```
# Look at missing data rows

missing_occ <- Occupation8 %>%
  filter(is.na(occupation_complexity))

## Retired people with no job history recorded

Occupation_sum2 <- Occupation8 %>%
  group_by(occupation_complexity) %>%
  summarise(count = n(),
            .groups='drop') %>%
  mutate(freq = count / sum(count))

## Higher = less complex

### ========================== Covariates ====================================================

Covaraites <- raw_data %>%
  select(id,c(any_of(covariate))) %>%
  filter(id %in% edu_dem_data_available$id)

check <- Covaraites %>%
  filter(is.na(age))

Age_sum <- Covaraites %>%
  group_by(ageband) %>%
  summarise(count = n(),
            .groups='drop') %>%
  mutate(freq = count / sum(count))

Sex_sum <- Covaraites %>%
  group_by(sex) %>%
  summarise(count = n(),
            .groups='drop') %>%
  mutate(freq = count / sum(count))


#APOE_sum <- Covaraites %>%
# group_by(apoe) %>%
#summarise(count = n(),
#          .groups='drop') %>%
#mutate(freq = count / sum(count))
#Lots of missing data - don't include

Ethnicity_sum <-  Covaraites %>%
  group_by(ethnicity) %>%
  summarise(count = n(),
            .groups='drop') %>%
  mutate(freq = count / sum(count))

## Group into white and not white

### Clean covariates

Covariates2 <- Covaraites %>%
  mutate(agecat = case_when(age > 34 & age < 50 ~ -1, # -1 = under 50
                            age > 49 & age < 55 ~ 0, # 0 = 50-54
                            age > 54 & age < 60 ~ 1, # 1 = 55-59
                            age > 59 & age < 65 ~ 2,#  2 = 60-64
                            age > 64 ~ 3), # 5 = 65-69
         ethnicitycat = case_when(ethnicity==1 ~ 0,
                                  ethnicity %in% c(2,3,4,5,6) ~ 1))

Ethnicity_sum2 <-  Covariates2 %>%
  group_by(ethnicitycat) %>%
  summarise(count = n(),
            .groups='drop') %>%
  mutate(freq = count / sum(count))

Age_sum2 <-  Covariates2 %>%
  group_by(agecat) %>%
  summarise(count = n(),
            .groups='drop') %>%
  mutate(freq = count / sum(count))


check <- Covariates2 %>%
  filter(is.na(agecat))


##  ----------- Collate into final dataset ---------------------------------------------

data_for_analysis <- edu_dem_data_available %>% left_join(Dementia,
                                                         by='id')%>% left_join(Education2,
                                                                                    by = 'id') %>% left_join(Health_outcomes,
                                                                                                              by ='id') %>%
left_join(Health_behaviours3,
                                                                                                                                        by =
'id') %>% left_join(Wealth,

by = 'id') %>% left_join(Occupation8,

by='id') %>% left_join(Isolation,

by='id') %>% left_join(Covariates2,

by='id') %>%
  filter(agecat != -1 ) %>%
  mutate(edu_cat_c = case_when(edu_cat2 == 0 ~ "GSCE education or lower",
                               edu_cat2 == 1 ~ "Completed secondary education",
                               edu_cat2 == 2 ~ "Higher education"),
         sexc = case_when(sex == 0 ~ "Female", sex == 1 ~ "Male",TRUE ~ as.character(sex)),
         age_cat_c = case_when(agecat == 0 ~ "50-54",
```

```
                                agecat == 1 ~ "55-59",
                                agecat == 2 ~ "60-64",
                                agecat == 3 ~ "65-69"),
         ethnicity_cat_c = case_when(ethnicitycat == 0 ~ "Caucasian",
                                      ethnicitycat == 1 ~ "Not Caucasian"),
         ho_summary_c_anyna_na = case_when(ho_summary_anyna_na==0 ~ "Excellent health",
                                            ho_summary_anyna_na==1 ~ "Good health",
                                            ho_summary_anyna_na==2 ~ "Intermediate health",
                                            ho_summary_anyna_na==3 ~ "Poor health"),
         ho_summary_c_adu = case_when(ho_summary_adu==0 ~ "Excellent health",
                                       ho_summary_adu==1 ~ "Good health",
                                       ho_summary_adu==2 ~ "Intermediate health",
                                       ho_summary_adu==3 ~ "Poor health"),
         hb_summary_c_anyna_na = case_when(healthbehaviourcat_anyna_na==0 ~ "Favourable",
                                            healthbehaviourcat_anyna_na==1 ~ "Intermediate",
                                            healthbehaviourcat_anyna_na==2 ~ "Unfavourable"),
         hb_summary_c_adu = case_when(healthbehaviourcat_adu==0 ~ "Favourable",
                                       healthbehaviourcat_adu==1 ~ "Intermediate",
                                       healthbehaviourcat_adu==2 ~ "Unfavourable"),
         avg_income_c = case_when(avg_household_income_c==1 ~ "< 18,000",
                                   avg_household_income_c==2 ~ "18,000 - 30,999",
                                   avg_household_income_c==3 ~ "31,000 - 51,999",
                                   avg_household_income_c==4 ~ "52,000 - 100,000",
                                   avg_household_income_c==5 ~ ">100,000"),
         occupation_complex_c = case_when(occupation_complexity==0 ~ "Managerial, proffesional and associate proffesional",
                                           occupation_complexity==1 ~ "Administrative, trades and caring proffesions",
                                           occupation_complexity==2 ~ "Sales, process, plant and machine operatives and elementary
occupations",
                                           occupation_complexity==3 ~ "Unemployed"),
         social_c = case_when(social==1 ~ "Socially isolated",
                               social==0 ~ "Not socially isolated"),
         alldem_c = case_when(alldem==1 ~ "Dementia",
                               alldem==0 ~ "No dementia"))

Age_check <-  data_for_analysis %>%
  group_by(agecat) %>%
  summarise(count = n(),
            .groups='drop') %>%
  mutate(freq = count / sum(count))

## Set up ordinal variables
data_for_analysis$edu_cat_c <- factor(data_for_analysis$edu_cat_c, order = TRUE, levels =c('GSCE education or lower', 'Completed
secondary education', 'Higher education'))
data_for_analysis$age_cat_c <- factor(data_for_analysis$age_cat_c, order = TRUE, levels =c('50-54', '55-59', '60-64','65-69'))
data_for_analysis$ho_summary_c_anyna_na <- factor(data_for_analysis$ho_summary_c_anyna_na, order = TRUE, levels =c('Excellent
health', 'Good health', 'Intermediate health','Poor health'))
data_for_analysis$ho_summary_c_adu <- factor(data_for_analysis$ho_summary_c_adu, order = TRUE, levels =c('Excellent health', 'Good
health', 'Intermediate health','Poor health'))
data_for_analysis$hb_summary_c_anyna_na <- factor(data_for_analysis$hb_summary_c_anyna_na, order = TRUE, levels =c('Favourable',
'Intermediate', 'Unfavourable'))
data_for_analysis$hb_summary_c_adu <- factor(data_for_analysis$hb_summary_c_adu, order = TRUE, levels =c('Favourable',
'Intermediate', 'Unfavourable'))
data_for_analysis$avg_income_c <- factor(data_for_analysis$avg_income_c, order = TRUE, levels =c('< 18,000', '18,000 - 30,999',
'31,000 - 51,999', '52,000 - 100,000','>100,000'))
data_for_analysis$occupation_complex_c <- factor(data_for_analysis$occupation_complex_c, order = TRUE, levels =c('Managerial,
proffesional and associate proffesional', 'Administrative, trades and caring proffesions', 'Sales, process, plant and machine
operatives and elementary occupations','Unemployed'))
#data_for_analysis$social_c <- factor(data_for_analysis$social_c, order = TRUE, levels =c('Not socially isolated', 'Socially
isolated'))
#data_for_analysis$alldem_c <- factor(data_for_analysis$alldem_c, order = TRUE, levels =c('No dementia', 'Dementia'))


## Label variables
attr(data_for_analysis$edu_cat_c, "label") <- "Education category"
attr(data_for_analysis$sexc, "label") <- "Sex"
attr(data_for_analysis$age_cat_c, "label") <- "Age category"
attr(data_for_analysis$ethnicity_cat_c, "label") <- "Ethnicity category"
attr(data_for_analysis$ho_summary_c_adu, "label") <- "Health category - Available data used"
attr(data_for_analysis$ho_summary_c_anyna_na, "label") <- "Health category - All missing values excluded"
attr(data_for_analysis$hb_summary_c_adu, "label") <- "Health behaviour category - Available data used"
attr(data_for_analysis$hb_summary_c_anyna_na, "label") <- "Health behaviour category - All missing values excluded"
attr(data_for_analysis$avg_income_c, "label") <- "Income category"
attr(data_for_analysis$occupation_complex_c, "label") <- "Occupational complexity"
attr(data_for_analysis$social_c, "label") <- "Social isolation"
attr(data_for_analysis$alldem_c, "label") <- "Dementia"

## Save data for next step
#save(data_for_analysis, file = "data_for_analysis_missinginc.RData")


# Missing Data Investigations - (Code 2) ####################################

## ---------- Set up ---------------------------------------------------------------------------------
#

library(summarytools)
library(tidyverse)
library("writexl")
library(qwraps2)
library(naniar)
library(ggplot2)
library(finalfit)
library(haven)
library(dplyr)
library(dtplyr)
library(broom)
library(jtools )
library(sjPlot)

setwd("/Volumes/GroupFolders/MHS_Naaheed_UKB/Katherine Taylor")
load("data_for_analysis_missinginc.RData")

data_refined <- data_for_analysis %>%
dplyr::select(edu_cat_c,sexc,age_cat_c,ethnicity_cat_c,ho_summary_c_anyna_na,ho_summary_c_adu,hb_summary_c_anyna_na,hb_summary_c_adu,a
```

```
## --------- Basic characteristics ------------------------------------------------------------

samplecharacteristics <- as.data.frame(dfSummary(data_refined, max.string.width = 500)) %>%
  dplyr::select(c(3,4,5,9))

write_xlsx(samplecharacteristics,"/Volumes/GroupFolders/Katherine Taylor/Results/complete_case_sample_c.xlsx")

## --------- Patterns of missing data ---------------------------------------------------------

gg_miss_upset(data_refined,
              nsets = 10,
              nintersects = 15)

# Missing data and demenita

gg_miss_var(data_refined,
            facet = alldem_c,
            show_pct = TRUE)

gg_miss_fct(x = data_refined, fct = alldem_c)

# Missing data and education

explanatory = c( "sexc","age_cat_c",  "ethnicity_cat_c", "ho_summary_c_anyna_na",
                 "ho_summary_c_adu","hb_summary_c_anyna_na","hb_summary_c_adu", "avg_income_c",
                 "occupation_complex_c", "social_c","alldem_c","townsend")
dependent = "edu_cat_c"

#data_refined %>%
# missing_pairs(dependent,explanatory)

data_refined %>%
  missing_compare(dependent, explanatory) %>%
  knitr::kable(row.names=FALSE, align = c("l", "l", "r", "r", "r"))

gg_miss_fct(x = data_refined, fct = edu_cat_c)


##  --------- Occupational complexity ---------------------------------------------------------


explanatory = c( "sexc","age_cat_c",  "ethnicity_cat_c", "ho_summary_c_anyna_na",
                 "ho_summary_c_adu","hb_summary_c_anyna_na","hb_summary_c_adu", "avg_income_c",
                 "social_c","edu_cat_c","alldem_c","townsend")
dependent = "occupation_complex_c"

#data_refined %>%
# missing_pairs(dependent,explanatory)


data_refined %>%
  missing_compare(dependent, explanatory) %>%
  knitr::kable(row.names=FALSE, align = c("l", "l", "r", "r", "r"))


## Linear regression

### Using derived variables where any missing data results in a missing observation overall


# Create missing occupational complexity variable

data_refined2 <- data_refined %>%
  mutate(occupation_missing = case_when(is.na(occupation_complex_c) ~ 1,
                                        TRUE ~ 0))

dataforreg1 <- data_refined2 %>%
  select(-c(6,8,10)) %>%
  mutate(sexc = as.factor(sexc),
         ethnicity_cat_c = as.factor(ethnicity_cat_c)) %>%
  na.omit()


model <- glm(occupation_missing ~ edu_cat_c + sexc + age_cat_c + ethnicity_cat_c + ho_summary_c_anyna_na + hb_summary_c_anyna_na +
avg_income_c + social_c + townsend ,family=binomial(link='logit'), data=dataforreg1)
summary(model)

tab_model(model, auto.label = TRUE)


### Using derived variables where all available data is used

#Create missing occupational complexity variable

dataforreg2 <- data_refined2 %>%
  select(-c(5,7,10)) %>%
  mutate(sexc = as.factor(sexc),
         ethnicity_cat_c = as.factor(ethnicity_cat_c)) %>%
  na.omit()

model2 <- glm(occupation_missing ~ edu_cat_c + sexc + age_cat_c + ethnicity_cat_c + ho_summary_c_adu + hb_summary_c_adu +
avg_income_c + social_c + townsend ,family=binomial(link='logit'), data=dataforreg2)
#summary(model)

tab_model(model2, auto.label = TRUE)


## --------- Income ---------------------------------------------------------------------------

explanatory = c( "sexc","age_cat_c",  "ethnicity_cat_c", "ho_summary_c_anyna_na",
                 "ho_summary_c_adu","hb_summary_c_anyna_na","hb_summary_c_adu", "occupation_complex_c",
                 "social_c","edu_cat_c","alldem_c","townsend")
dependent = "avg_income_c"
```

```
#data_refined %>%
# missing_pairs(dependent,explanatory)

data_refined %>%
  missing_compare(dependent, explanatory) %>%
  knitr::kable(row.names=FALSE, align = c("l", "l", "r", "r", "r"))


## Linear regression

### Using derived variables where any missing data results in a missing observation overall

#Create missing income variable

data_refined3 <- data_refined %>%
  mutate(income_missing = case_when(is.na(avg_income_c) ~ 1,
                                    TRUE ~ 0))

dataforreg3 <- data_refined3 %>%
  select(-c(6,8,9)) %>%
  mutate(sexc = as.factor(sexc),
         ethnicity_cat_c = as.factor(ethnicity_cat_c),
         alldem_c = as.factor(alldem_c),
         social_c = as.factor(social_c)) %>%
  na.omit()

model3 <- glm(income_missing ~ edu_cat_c + sexc + age_cat_c + ethnicity_cat_c + ho_summary_c_anyna_na + hb_summary_c_anyna_na +
occupation_complex_c + social_c + townsend ,family=binomial(link='logit'), data=dataforreg3)
#summary(model3)

tab_model(model3, auto.label = TRUE)


### Using derived variables where all available data is used


#Create missing income variable

dataforreg4 <- data_refined3 %>%
  select(-c(5,7,9)) %>%
  mutate(sexc = as.factor(sexc),
         ethnicity_cat_c = as.factor(ethnicity_cat_c),
         alldem_c = as.factor(alldem_c),
         social_c = as.factor(social_c)) %>%
  na.omit()


model4 <- glm(income_missing ~ edu_cat_c + sexc + age_cat_c + ethnicity_cat_c + ho_summary_c_adu + hb_summary_c_adu +
occupation_complex_c + social_c + townsend ,family=binomial(link='logit'), data=dataforreg4)
#(model4)

tab_model(model4, auto.label = TRUE)

## --------- Health Behaviour ---------------------------------------------------------------------

# Using derived variables where any missing data results in a missing observation overall

explanatory = c( "sexc","age_cat_c",  "ethnicity_cat_c", "ho_summary_c_anyna_na",
                 "ho_summary_c_adu","hb_summary_c_adu", "avg_income_c",
                 "social_c","edu_cat_c","alldem_c","occupation_complex_c","townsend")
dependent = "hb_summary_c_anyna_na"

data_refined %>%
  missing_compare(dependent, explanatory) %>%
  knitr::kable(row.names=FALSE, align = c("l", "l", "r", "r", "r"))

## Linear regression

### Using derived variables where any missing data results in a missing observation overall

#Create missing health behaviour variable

data_refined5 <- data_refined %>%
  mutate(hb_anyna_na_missing = case_when(is.na(hb_summary_c_anyna_na) ~ 1,
                                         TRUE ~ 0))

dataforreg5 <- data_refined5 %>%
  select(-c(7,8)) %>%
  mutate(sexc = as.factor(sexc),
         ethnicity_cat_c = as.factor(ethnicity_cat_c),
         alldem_c = as.factor(alldem_c),
         social_c = as.factor(social_c)) %>%
  na.omit()


model5 <- glm(hb_anyna_na_missing ~ edu_cat_c + sexc + age_cat_c + ethnicity_cat_c + ho_summary_c_anyna_na + occupation_complex_c +
social_c + townsend ,family=binomial(link='logit'), data=dataforreg5)
#summary(model3)

tab_model(model5, auto.label = TRUE)

# Multiple Imputation - (Code 3) ############################################################

## -------- Set up ---------------------------------------------------------------------

library(dplyr)
library(mice)
library(lattice)
library(data.table)
library(ggplot2)
library(lava)

setwd("/Volumes/GroupFolders/MHS_Naaheed_UKB/Katherine Taylor")
```

```
load("data_for_analysis_missinginc.RData")

depression <- data_for_analysis %>%
  select(c(1,11))
save(depression, file = "depression.RData")


data_refined <- data_for_analysis %>%
select(edu_cat_c,sexc,age_cat_c,ethnicity_cat_c,ho_summary_c_anyna_na,ho_summary_c_adu,hb_summary_c_anyna_na,hb_summary_c_adu,avg_inco

data_for_mi <- data_refined %>%
  mutate(ho_summary = ho_summary_c_anyna_na,
         hb_summary = hb_summary_c_anyna_na) %>%
  select(-c(5:8))

# Set up variable types

data_for_mi$ho_summary <- factor(data_for_mi$ho_summary, order = TRUE, levels =c('Excellent health', 'Good health', 'Intermediate
health','Poor health'))
data_for_mi$hb_summary <- factor(data_for_mi$hb_summary, order = TRUE, levels =c('Favourable', 'Intermediate', 'Unfavourable'))

data_for_mi$ethnicity_cat_c <- factor(data_for_mi$ethnicity_cat_c)

data_for_mi$social_c <- factor(data_for_mi$social_c)
data_for_mi$alldem_c <- factor(data_for_mi$alldem_c)
data_for_mi$sexc <- factor(data_for_mi$sexc)

## -------- Run the mice code with 0 iterations----------------------------------------

imp <- mice(data_for_mi, maxit=0)

# Extract predictorMatrix and methods of imputation

predM <- imp$predictorMatrix
meth <- imp$method

meth

# Manually change imputation method where required

meth["ethnicity_cat_c"] <- "polyreg"
meth["social_c"] <- "logreg"

meth

## -------- Set up imputation -------------------------------------------------------

imp2 <- mice(data_for_mi, maxit = 10, m = 20,
             predictorMatrix = predM,
             method = meth, print =  FALSE, seed = 13)

# Get datasets into long form
imp_long <- mice::complete(imp2, action="long", include = TRUE)


imp_check <- mice::complete(imp2, 1)
imp_check <- mice::complete(imp2, 20)

save(imp2,  file ="imputed_data.Rdata")
save(imp_long, file = "imputed_data_long.Rdata")

load("imputed_data.Rdata")
load("imputed_data_long.Rdata")

complete_case_data <- cc(imp2)

save(complete_case_data, file = "complete_case_data.Rdata")


head(imp2$imp$occupation_complex_c)

## --------  Compare imputed and complete case data ----------------------------------------

## Basic plots
plot(imp2, layout = c(1,2))
densityplot(imp2)

### Occupation
occupatation <- rbind(data.table(occupation = unlist(imp2$imp$occupation_complex_c), imputed = TRUE),
                      data.table(occupation = na.omit(data_for_mi$occupation_complex_c), imputed = FALSE))

ggplot(occupatation, aes(x=as.factor(occupation), fill=as.factor(imputed)))+
  geom_bar(aes( y=..count../tapply(..count.., ..fill.. ,sum)[..fill..]), position="dodge" ) +
  geom_text(aes( y=..count../tapply(..count.., ..fill.. ,sum)[..fill..], label=scales::percent(..count../tapply(..count.., ..fill..
,sum)[..fill..]) ),
          stat="count", position=position_dodge(0.9), vjust=-0.5)+
  ylab('Percent of Occupation, %') +
  scale_y_continuous(labels = scales::percent) +
  theme(axis.text.x = element_text(angle = 15, vjust = 0.5))

### Income
income <- rbind(data.table(income = unlist(imp2$imp$avg_income_c), imputed = TRUE),
                data.table(income = na.omit(data_for_mi$avg_income_c), imputed = FALSE))

ggplot(income, aes(x=as.factor(income), fill=as.factor(imputed)))+
  geom_bar(aes( y=..count../tapply(..count.., ..fill.. ,sum)[..fill..]), position="dodge" ) +
  geom_text(aes( y=..count../tapply(..count.., ..fill.. ,sum)[..fill..], label=scales::percent(..count../tapply(..count.., ..fill..
,sum)[..fill..]) ),
          stat="count", position=position_dodge(0.9), vjust=-0.5)+
  ylab('Percent of Income categories, %') +
  scale_y_continuous(labels = scales::percent) +
  theme(axis.text.x = element_text(angle = 15, vjust = 0.5))
```

```
### Health behaviours
healthbehaviour <- rbind(data.table(hb = unlist(imp2$imp$hb_summary), imputed = TRUE),
                         data.table(hb = na.omit(data_for_mi$hb_summary), imputed = FALSE))

ggplot(healthbehaviour, aes(x=as.factor(hb), fill=as.factor(imputed)))+
  geom_bar(aes( y=..count../tapply(..count.., ..fill.. ,sum)[..fill..]), position="dodge" ) +
  geom_text(aes( y=..count../tapply(..count.., ..fill.. ,sum)[..fill..], label=scales::percent(..count../tapply(..count.., ..fill..
,sum)[..fill..]) ),
            stat="count", position=position_dodge(0.9), vjust=-0.5)+
  ylab('Percent of Income categories, %') +
  scale_y_continuous(labels = scales::percent) +
  theme(axis.text.x = element_text(angle = 15, vjust = 0.5))

stripplot(imp2, hb_summary???.imp, pch=20, cex=2)


# Recategorisation of variables - (Code 4) ############################################
# Decided to make varaibles binary following multiple imputation

# ------------- Set up -----------------------------------------------------------------------------------
#

library(haven)
library(dplyr)
library(dtplyr)
library(finalfit)
library(summarytools)
library(matrixStats)
library(hablar)
library(naniar)
library(mice)

#Set working directory
setwd("/Volumes/GroupFolders/MHS_Naaheed_UKB/Katherine Taylor")
getwd()


# Data loading

load("imputed_data_long.RData")
load("data_for_analysis_missinginc.RData")
load("depression.RData")

## ------------- Data refinement -----------------------------------------------------------------

# Make variables binary
imp_long2 <- imp_long %>%
  mutate(edu_cat_b = case_when(edu_cat_c == "GSCE education or lower" ~ "Education up 16",
                               TRUE ~ "Education after 16"),
         income_b = case_when(avg_income_c %in% c("< 18,000","18,000 - 30,999") ~ "< 31,000",
                              TRUE ~ "> 30,999"),
         occupation_b = case_when(occupation_complex_c == "Managerial, proffesional and associate proffesional" ~ "Manegerial &
proffesional",
                                  TRUE ~ "Non mangerial or proffesional occupations"),
         ho_summary_b = case_when(ho_summary %in% c("Good health","Excellent health") ~ "Good/Excellent Health",
                                  TRUE ~ "Intermediate/Poor health"),
         hb_summary_b = case_when(hb_summary == "Favourable" ~ "Favourable",
                                  TRUE ~ "Unfavourable"))

# Join on continous age

data_for_analysis$rownum <- seq.int(nrow(data_for_analysis))

imp_long3 <- imp_long2 %>%
  left_join(data_for_analysis %>%
              dplyr::select(age,rownum),
            by = c(".id"="rownum"))


# Join on depression
depression$rownum <- seq.int(nrow(depression))
imp_long3$rownum <- seq.int(nrow(imp_long3))


imp_long3 <- imp_long3 %>%
  left_join(depression, by = c('rownum'='rownum'))


# Dataset finalizing

imp_fixed<-as.mids(imp_long3)

imputed_datasets <- mice::complete(imp_fixed, c(1:20))

complete_case_data_fix <- cc(imp_fixed)

save(complete_case_data_fix, file = "complete_case_data_fix.Rdata")
save(imp_fixed, file = "imputed_data_fix.Rdata")
save(imp_long3, file = "imp_long_fix.Rdata")


# Descriptive analysis - (Code 5) ######################################

## --------- Set up  ------------------------------------------------------------------------------

library(summarytools)
library(tidyverse)
library("writexl")
library(qwraps2)
library(ggpubr)
library(plotrix)

setwd("S:/MHS_Naaheed_UKB/Katherine Taylor")
load("complete_case_data_fix.Rdata")
```

```
load("depression.RData")

complete_case_data <- complete_case_data_fix

data_refined <- complete_case_data %>%
dplyr::select(edu_cat_b,sexc,age,ethnicity_cat_c,ho_summary_b,hb_summary_b,income_b,occupation_b,social_c,alldem_c,depression2)

## --------- Sample Characteristics ---------------------------------------------------------------------------------

samplecharacteristics <- as.data.frame(dfSummary(data_refined, max.string.width = 500)) %>%
  dplyr::select(c(3,4,5))

write_xlsx(samplecharacteristics, "/Volumes/GroupFolders/MHS_Naaheed_UKB/Katherine
Taylor/Results/Sample_Characteristics_completecase_v2.xlsx")

samplecharacteristics2 <- summary_table(data_refined)

## --------- Associations with Education ------------------------------------------------------

## Cross-tabulations

ctable(data_refined$alldem_c, data_refined$edu_cat_b,
       prop = "c", chisq = TRUE, headings = FALSE, totals = FALSE,dnn = c('Dementia', ''))

ctable(data_refined$sexc, data_refined$edu_cat_b,
       prop = "c", chisq = TRUE, headings = FALSE, totals = FALSE, dnn = c('Sex', ''))

ctable(data_refined$ethnicity_cat_c, data_refined$edu_cat_b,
       prop = "c", chisq = TRUE, headings = FALSE, totals = FALSE, dnn = c('Ethnicity', ''))

ctable(data_refined$ho_summary_b, data_refined$edu_cat_b,
       prop = "c", chisq = TRUE, headings = FALSE, totals = FALSE, dnn = c('Health Outcomes', ''))

ctable(data_refined$hb_summary_b, data_refined$edu_cat_b,
       prop = "c", chisq = TRUE, headings = FALSE, totals = FALSE,dnn = c('Health Behaviours', ''))

ctable(data_refined$income_b, data_refined$edu_cat_b,
       prop = "c", chisq = TRUE, headings = FALSE, totals = FALSE,dnn = c('Income', ''))

ctable(data_refined$occupation_b, data_refined$edu_cat_b,
       prop = "c", chisq = TRUE, headings = FALSE, totals = FALSE,dnn = c('Occupational Complexity', ''))

ctable(data_refined$social_c, data_refined$edu_cat_b,
       prop = "c", chisq = TRUE, totals = FALSE, dnn = c('Social Isolation', ''))

ctable(data_refined$income_b, data_refined$occupation_b,
       prop = "c", chisq = TRUE, totals = FALSE, dnn = c('Income', ''))

data_refined %>%  select(age, edu_cat_b) %>% group_by(edu_cat_b) %>%
        summarise_each(funs(mean,sd,std.error))

## Dementia

data_refined %>%
  ggplot() +
  aes(x = edu_cat_b, fill = alldem_c) +
  geom_bar(position="fill") +
  xlab("Education")+
  ylab("No. Participants") +
  labs(fill = "Dementia Status") +
  theme(axis.text.x = element_text(angle = 15, vjust = 0.5),
        panel.border = element_blank(),
        panel.grid.major = element_blank(),
        panel.grid.minor = element_blank())


## Sex

data_refined %>%
  ggplot() +
  aes(x = edu_cat_b, fill = sexc) +
  geom_bar(position="fill") +
  xlab("Education")+
  ylab("No. Participants") +
  labs(fill = "Sex") +
  theme(axis.text.x = element_text(angle = 15, vjust = 0.5),
        panel.border = element_blank(),
        panel.grid.major = element_blank(),
        panel.grid.minor = element_blank())

## Age

theme_set(theme_pubclean())

data_refined %>%
  ggplot() +
  aes(x = age )+ geom_freqpoly( aes(color = edu_cat_b),
                               bins = 19, size = 1.5) +
  scale_color_manual(values = c("#00AFBB", "#E7B800"))


## Ethnicity

data_refined %>%
  ggplot() +
  aes(x = edu_cat_b, fill = ethnicity_cat_c) +
  geom_bar(position="fill") +
  xlab("Education")+
  ylab("No. Participants") +
  labs(fill = "Ethnicity") +
  theme(axis.text.x = element_text(angle = 15, vjust = 0.5),
        panel.border = element_blank(),
        panel.grid.major = element_blank(),
        panel.grid.minor = element_blank())
```

```
## Health Outcomes
data_refined %>%
  ggplot() +
  aes(x = edu_cat_b, fill = ho_summary_b) +
  geom_bar(position="fill") +
  xlab("Education")+
  ylab("No. Participants") +
  labs(fill = "Health Outcomes") +
  theme(axis.text.x = element_text(angle = 15, vjust = 0.5),
        panel.border = element_blank(),
        panel.grid.major = element_blank(),
        panel.grid.minor = element_blank())


## Health Behaviours
data_refined %>%
  ggplot() +
  aes(x = edu_cat_b, fill = hb_summary_b) +
  geom_bar(position="fill") +
  xlab("Health Behaviours")+
  ylab("No. Participants") +
  labs(fill = "Ethnicity") +
  theme(axis.text.x = element_text(angle = 15, vjust = 0.5),
        panel.border = element_blank(),
        panel.grid.major = element_blank(),
        panel.grid.minor = element_blank())


## Income
data_refined %>%
  ggplot() +
  aes(x = edu_cat_b, fill = income_b) +
  geom_bar(position="fill") +
  xlab("Education")+
  ylab("No. Participants") +
  labs(fill = "Income") +
  theme(axis.text.x = element_text(angle = 15, vjust = 0.5),
        panel.border = element_blank(),
        panel.grid.major = element_blank(),
        panel.grid.minor = element_blank())


## Occupational Complexity

data_refined %>%
  ggplot() +
  aes(x = edu_cat_b, fill = occupation_b) +
  geom_bar(position="fill") +
  xlab("Education")+
  ylab("No. Participants") +
  labs(fill = "Occupational Complexity") +
  theme(axis.text.x = element_text(angle = 15, vjust = 0.5),
        panel.border = element_blank(),
        panel.grid.major = element_blank(),
        panel.grid.minor = element_blank())


## Social Isolation
data_refined %>%
  ggplot() +
  aes(x = edu_cat_b, fill = social_c) +
  geom_bar(position="fill") +
  xlab("Education")+
  ylab("No. Participants") +
  labs(fill = "Social Isolation") +
  theme(axis.text.x = element_text(angle = 15, vjust = 0.5),
        panel.border = element_blank(),
        panel.grid.major = element_blank(),
        panel.grid.minor = element_blank())


## --------- Associations with Dementia ------------------------------------------------------


## Cross-tabulations
ctable(data_refined$edu_cat_b, data_refined$alldem_c,
       prop = "c", chisq = TRUE, headings = FALSE, totals = FALSE,dnn = c('Dementia', ''),
)
ctable(data_refined$sexc, data_refined$alldem_c,
       prop = "c", chisq = TRUE, headings = FALSE, totals = FALSE, dnn = c('Sex', ''),)

ctable(data_refined$ethnicity_cat_c, data_refined$alldem_c,
       prop = "c", chisq = TRUE, headings = FALSE, totals = FALSE, dnn = c('Ethnicity', ''),)

ctable(data_refined$ho_summary_b, data_refined$alldem_c,
       prop = "c", chisq = TRUE, headings = FALSE, totals = FALSE, dnn = c('Health Outcomes', ''),)

ctable(data_refined$hb_summary_b, data_refined$alldem_c,
       prop = "c", chisq = TRUE, headings = FALSE, totals = FALSE,dnn = c('Health Behaviours', ''),)

ctable(data_refined$income_b, data_refined$alldem_c,
       prop = "c", chisq = TRUE, headings = FALSE, totals = FALSE,dnn = c('Income', ''),)

ctable(data_refined$occupation_b, data_refined$alldem_c,
       prop = "c", chisq = TRUE, headings = FALSE, totals = FALSE,dnn = c('Occupational Complexity', ''),)

ctable(data_refined$social_c, data_refined$alldem_c,
       prop = "c", chisq = TRUE, totals = FALSE, dnn = c('Social Isolation', ''), headings = FALSE)

print(data_refined %>%  select(age, alldem_c) %>% group_by(alldem_c) %>%
        summarise_each(funs(mean,sd,std.error)))
```

```
## Education

data_refined %>%
  ggplot() +
  aes(x = alldem_c, fill = edu_cat_b) +
  geom_bar(position="fill") +
  xlab("Dementia Status")+
  ylab("No. Participants") +
  labs(fill = "Education") +
  theme(panel.border = element_blank(),
        panel.grid.major = element_blank(),
        panel.grid.minor = element_blank())


## Sex

data_refined %>%
  ggplot() +
  aes(x = alldem_c, fill = sexc) +
  geom_bar(position="fill") +
  xlab("Sex")+
  ylab("No. Participants") +
  labs(fill = "Sex") +
  theme(panel.border = element_blank(),
        panel.grid.major = element_blank(),
        panel.grid.minor = element_blank())


## Age

theme_set(theme_pubclean())

data_refined %>%
  ggplot() +
  aes(x = age )+ geom_freqpoly( aes(color = alldem_c),
                                bins = 19, size = 1.5) +
  scale_color_manual(values = c("#00AFBB", "#E7B800"))


## Ethnicity

data_refined %>%
  ggplot() +
  aes(x = alldem_c, fill = ethnicity_cat_c) +
  geom_bar(position="fill") +
  xlab("Ethnicity")+
  ylab("No. Participants") +
  labs(fill = "Sex") +
  theme(panel.border = element_blank(),
        panel.grid.major = element_blank(),
        panel.grid.minor = element_blank())


## Health Outcomes

data_refined %>%
  ggplot() +
  aes(x = alldem_c, fill = ho_summary_b) +
  geom_bar(position="fill") +
  xlab("Health Outcomes")+
  ylab("No. Participants") +
  labs(fill = "Sex") +
  theme(panel.border = element_blank(),
        panel.grid.major = element_blank(),
        panel.grid.minor = element_blank())


## Health Behaviours

data_refined %>%
  ggplot() +
  aes(x = alldem_c, fill = hb_summary_b) +
  geom_bar(position="fill") +
  xlab("Health Behaviours")+
  ylab("No. Participants") +
  labs(fill = "Sex") +
  theme(panel.border = element_blank(),
        panel.grid.major = element_blank(),
        panel.grid.minor = element_blank())


## Income

data_refined %>%
  ggplot() +
  aes(x = alldem_c, fill = income_b) +
  geom_bar(position="fill") +
  xlab("Income")+
  ylab("No. Participants") +
  labs(fill = "Sex") +
  theme(panel.border = element_blank(),
        panel.grid.major = element_blank(),
        panel.grid.minor = element_blank())


## Occupational Complexity

data_refined %>%
  ggplot() +
  aes(x = alldem_c, fill = occupation_b) +
  geom_bar(position="fill") +
  xlab("Dementia Status")+
  ylab("No. Participants") +
```

```
  labs(fill = "Sex") +
  theme(panel.border = element_blank(),
        panel.grid.major = element_blank(),
        panel.grid.minor = element_blank())


## Social Isolation

data_refined %>%
  ggplot() +
  aes(x = alldem_c, fill = social_c) +
  geom_bar(position="fill") +
  xlab("Occupational Complexity")+
  ylab("No. Participants") +
  labs(fill = "Sex") +
  theme(panel.border = element_blank(),
        panel.grid.major = element_blank(),
        panel.grid.minor = element_blank())

test <- ctable(x= data_refined$edu_cat_b,
               y= data_refined$alldem_c,
               chisq = TRUE)


#Logistic regression - (Code 6) ########################################################

## --------- Set up -----------------------------------------------------------------------------------

install.packages('xlsx')
install.packages('mitools')

library(tidyverse)
library(mice)
library(mitools)
library(norm)
library("writexl")
library(xlsx)


#setwd("S:/MHS_Naaheed_UKB/Katherine Taylor")
setwd("/Volumes/GroupFolders/MHS_Naaheed_UKB/Katherine Taylor")

load("imputed_data_fix.Rdata")
load("imp_long_fix.Rdata")

imp_long3$alldem_c <- factor(imp_long3$alldem_c, order = TRUE, levels =c('No dementia', 'Dementia'))

### Un-order factors to observe threshold effects
imp_long3$hb_summary_b <- factor(imp_long3$hb_summary_b, order = FALSE)
imp_long3$ho_summary_b <- factor(imp_long3$ho_summary_b, order = FALSE)
imp_long3$income_b <- factor(imp_long3$income_b, order = TRUE, levels =c('< 31,000','> 30,999'))
imp_long3$income_b <- factor(imp_long3$income_b, order = FALSE)
imp_long3$occupation_b <- factor(imp_long3$occupation_b, order = FALSE)
imp_long3$social_c <- factor(imp_long3$social_c, order = FALSE)
imp_long3$edu_cat_b <- factor(imp_long3$edu_cat_b, order = TRUE, levels =c('Education up 16','Education after 16'))
imp_long3$edu_cat_b <- factor(imp_long3$edu_cat_b, order = FALSE)

#imp_long$age_cat_c <- factor(imp_long$age_cat_c, order = FALSE)
#imp_long$edu_cat_c <- factor(imp_long$edu_cat_c, order = FALSE)


imp_fix2<-as.mids(imp_long3)
str(imp_fix2)

## --------- Combined Models ------------------------------------------------------------------------

model1u <- with(imp_fix2,
                glm(alldem_c ~ edu_cat_b  + sexc + age + ethnicity_cat_c, family=binomial))
model1_sumu <- summary(pool(model1u),conf.int = TRUE, exponentiate = TRUE) %>%
  mutate_if(is.numeric, ~round(., 3))  %>%
  select(c(1,2,6,7,8))

covariate <- "Adjusted for Age, Sex & Ethnicity"
model1u_semf <- rbind(covariate, model1_sumu)

model2u <- with(imp_fix2,
                glm(alldem_c ~ edu_cat_b  + sexc + age + ethnicity_cat_c + ho_summary_b, family=binomial))
model2_sumu <- summary(pool(model2u),conf.int = TRUE, exponentiate = TRUE) %>%
  mutate_if(is.numeric, ~round(., 3))  %>%
  select(c(1,2,6,7,8))

covariate <- "+ Health Outcome"
model2u_semf <- rbind(covariate, model2_sumu)

#### Age, sex, ethnicity, health outcome, social isolation

model3u <- with(imp_fix2,
                glm(alldem_c ~ edu_cat_b  + sexc + age + ethnicity_cat_c + ho_summary_b + social_c, family=binomial))
model3_sumu <- summary(pool(model3u),conf.int = TRUE, exponentiate = TRUE) %>%
  mutate_if(is.numeric, ~round(., 3))  %>%
  select(c(1,2,6,7,8))

covariate <- "+ Health Outcome & Social Isolation"
model3_sumuf <- rbind(covariate, model3_sumu)

#### Age, sex, ethnicity, health outcome, social isolation, health behaviour

model4u <- with(imp_fix2,
                glm(alldem_c ~ edu_cat_b  + sexc + age + ethnicity_cat_c + ho_summary_b + social_c + hb_summary_b, family=binomial))
model4_sumu <- summary(pool(model4u),conf.int = TRUE, exponentiate = TRUE) %>%
  mutate_if(is.numeric, ~round(., 3)) %>%
  select(c(1,2,6,7,8))

covariate <- "+ Health Outcome, Social Isolation & Health Behaviour"
```

```
model4_sumuf <- rbind(covariate, model4_sumu)

#### Age, sex, ethnicity, health outcome, social isolation, wealth, health behaviour

model5u <- with(imp_fix2,
                glm(alldem_c ~ edu_cat_b  + sexc + age + ethnicity_cat_c + ho_summary_b + social_c + income_b + hb_summary_b,
family=binomial))
model5_sumu <- summary(pool(model5u),conf.int = TRUE, exponentiate = TRUE) %>%
  mutate_if(is.numeric, ~round(., 3)) %>%
  select(c(1,2,6,7,8))

covariate <- "+ Health Outcome, Social Isolation, Health Behaviour & Wealth"
model5_sumuf <- rbind(covariate, model5_sumu)

#### Age, sex, ethnicity, health outcome, social isolation, health behaviour, occupation

model6u <- with(imp_fix2,
                glm(alldem_c ~ edu_cat_b  + sexc + age + ethnicity_cat_c + ho_summary_b + social_c + hb_summary_b + occupation_b,
family=binomial))
model6_sumu <- summary(pool(model6u),conf.int = TRUE, exponentiate = TRUE) %>%
  mutate_if(is.numeric, ~round(., 3)) %>%
  select(c(1,2,6,7,8))

covariate <- "+ Health Outcome, Social Isolation, Health Behaviour & Occupation"
model6_sumuf <- rbind(covariate, model6_sumu)

### Combined model

comb_med1 <- rbind(model1u_semf, model2u_semf, model3_sumuf, model4_sumuf, model5_sumuf, model6_sumuf)
write.xlsx(comb_med1, "/Volumes/GroupFolders/MHS_Naaheed_UKB/Katherine Taylor/Results/Logistic_Regression_v2.xlsx", sheetName =
"Combined Mediators", append = TRUE)


# Complete case logistic regression - (Code 7) ################################################


## ----------- Set up ------------------------------------------------------------------------------

library(tidyverse)
library(mice)
library(mitools)
library(norm)
library("writexl")
library(xlsx)

#setwd("S:/MHS_Naaheed_UKB/Katherine Taylor")
setwd("/Volumes/GroupFolders/MHS_Naaheed_UKB/Katherine Taylor")

load("complete_case_data_fix.Rdata")

complete_case_data_fix$alldem_c <- factor(complete_case_data_fix$alldem_c, order = TRUE, levels =c('No dementia', 'Dementia'))

### Un-order factors to observe threshold effects
complete_case_data_fix$hb_summary_b <- factor(complete_case_data_fix$hb_summary_b, order = FALSE)
complete_case_data_fix$ho_summary_b <- factor(complete_case_data_fix$ho_summary_b, order = FALSE)
complete_case_data_fix$income_b <- factor(complete_case_data_fix$income_b, order = TRUE, levels =c('< 31,000','> 30,999'))
complete_case_data_fix$income_b <- factor(complete_case_data_fix$income_b, order = FALSE)
complete_case_data_fix$occupation_b <- factor(complete_case_data_fix$occupation_b, order = FALSE)
complete_case_data_fix$social_c <- factor(complete_case_data_fix$social_c, order = FALSE)
complete_case_data_fix$edu_cat_b <- factor(complete_case_data_fix$edu_cat_b, order = TRUE, levels =c('Education up 16','Education
after 16'))
complete_case_data_fix$edu_cat_b <- factor(complete_case_data_fix$edu_cat_b, order = FALSE)


## ----------- Combined Models -------------------------------------------------------------------

model1u <- glm(alldem_c ~ edu_cat_b  + sexc + age + ethnicity_cat_c, family=binomial(link='logit'), data=complete_case_data_fix)
model1_sumu <- exp(cbind(OR = coef(model1u), confint(model1u)))
covariate <- "Adjusted for Age, Sex & Ethnicity"
model1u_semf <- rbind(covariate, model1_sumu)

model2u <- glm(alldem_c ~ edu_cat_b  + sexc + age + ethnicity_cat_c + ho_summary_b, family=binomial(link='logit'),
data=complete_case_data_fix)
model2_sumu <- exp(cbind(OR = coef(model2u), confint(model2u)))
covariate <- "+ Health Outcome"
model2u_semf <- rbind(covariate, model2_sumu)

#### Age, sex, ethnicity, health outcome, social isolation

model3u <- glm(alldem_c ~ edu_cat_b  + sexc + age + ethnicity_cat_c + ho_summary_b + social_c, family=binomial(link='logit'),
data=complete_case_data_fix)
model3_sumu <- exp(cbind(OR = coef(model3u), confint(model3u)))

covariate <- "+ Health Outcome & Social Isolation"
model3_sumuf <- rbind(covariate, model3_sumu)

#### Age, sex, ethnicity, health outcome, social isolation, health behaviour

model4u <- glm(alldem_c ~ edu_cat_b  + sexc + age + ethnicity_cat_c + ho_summary_b + social_c + hb_summary_b,
family=binomial(link='logit'), data=complete_case_data_fix)
model4_sumu <- exp(cbind(OR = coef(model4u), confint(model4u)))

covariate <- "+ Health Outcome, Social Isolation & Health Behaviour"
model4_sumuf <- rbind(covariate, model4_sumu)

#### Age, sex, ethnicity, health outcome, social isolation, wealth, health behaviour

model5u <- glm(alldem_c ~ edu_cat_b  + sexc + age + ethnicity_cat_c + ho_summary_b + social_c + income_b + hb_summary_b,
family=binomial(link='logit'), data=complete_case_data_fix)
model5_sumu <- exp(cbind(OR = coef(model5u), confint(model5u)))

covariate <- "+ Health Outcome, Social Isolation, Health Behaviour & Wealth"
model5_sumuf <- rbind(covariate, model5_sumu)
```

```
#### Age, sex, ethnicity, health outcome, social isolation, health behaviour, occupation

model6u <- glm(alldem_c ~ edu_cat_b  + sexc + age + ethnicity_cat_c + ho_summary_b + social_c + hb_summary_b + occupation_b,
family=binomial, data=complete_case_data_fix)
model6_sumu <- exp(cbind(OR = coef(model6u), confint(model6u)))

covariate <- "+ Health Outcome, Social Isolation, Health Behaviour & Occupation"
model6_sumuf <- rbind(covariate, model6_sumu)

### Combined model

comb_med1 <- rbind(model1u_semf, model2u_semf, model3_sumuf, model4_sumuf, model5_sumuf, model6_sumuf)
write.xlsx(comb_med1, "/Volumes/GroupFolders/MHS_Naaheed_UKB/Katherine Taylor/Results/Logistic_Regression_cc_v2.xlsx", sheetName =
"Combined Mediators", append = TRUE)


# Mediation analysis - (Code 8) ##########################################################################################

## ----------- Set up ---------------------------------------------------------------------

setwd("/Volumes/GroupFolders/MHS_Naaheed_UKB/Katherine Taylor")
library(mice)
library(tidyverse)
library(mediation)
library(mitools)
library(miceadds)
library(diagram)

install.packages("Cairo")
library("Cairo")

load("imp_long_fix.RData")

imp_long3$edu_cat_b <- as.factor(imp_long3$edu_cat_b)

imp_long3$edu_dum <- case_when(imp_long3$edu_cat_b == "Education up 16" ~ 1,
                               TRUE ~ 0)

imp_long3$dem_dum <- case_when(imp_long3$alldem_c == "Dementia" ~ 1,
                               TRUE ~ 0)


#Set mediators as binary 0/1

imp_long3$income_dum <- case_when(imp_long3$income_b == "< 31,000" ~ 1,
                                  TRUE ~ 0)
imp_long3$healtho_dum <- case_when(imp_long3$ho_summary_b == "Intermediate/Poor health" ~ 1,
                                   TRUE ~ 0)
imp_long3$healthb_dum <- case_when(imp_long3$hb_summary_b == "Unfavourable" ~ 1,
                                   TRUE ~ 0)
imp_long3$occupationb_dum <- case_when(imp_long3$occupation_b == "Non mangerial or proffesional occupations" ~ 1,
                                       TRUE ~ 0)

imp_long3$socialb_dum <- case_when(imp_long3$social_c == "Socially isolated" ~ 1,
                                   TRUE ~ 0)


imp_long4 <- imp_long3 %>%
  dplyr::select(1,2,4,6,19,21:29)

imp_test <-as.mids(imp_long4)

completemice <- imp_test %>%
  mice::complete("all")

## ----------- Health Outcomes -----------------------------------------------------------------------------

data <- names(completemice)

# Run mediation analysis for each imputation seperately, then combine using the amelidiate command.
# Mediations command not used as we wished to adjust for different covariates on each pathway

healthoutcome <- lapply(completemice, FUN = function(data){

  out <- list()
  count <- 1

  ind <- glm(healtho_dum ~ edu_dum + sexc + age + ethnicity_cat_c, data = data,
             family=binomial(link="probit"))
  dir <- glm(dem_dum ~ edu_dum + healtho_dum + sexc + age + ethnicity_cat_c + healthb_dum + income_dum + occupationb_dum, data =
data,
             family=binomial(link="probit"))
  out <- mediate(ind, dir, sims=1000, treat="edu_dum",mediator="healtho_dum")
  summary(out)
  class(out) <- "mediations"
  return(out)
  # out
})


names <- letters[1:20]
names(healthoutcome) <- names

healthoutcome2 <- amelidiate(healthoutcome)
saveRDS(healthoutcome2, file = "Results/Healthoutmed.RData")

healthoutcomesum <- summary(healthoutcome2)
saveRDS(healthoutcomesum, file = "Results/Healthoutmedsum.RData")


jpeg(file="Results/CM_healthoutcome.jpeg")
plot(healthoutcome2)
dev.off()
```

```
#healthout <- readRDS("Results/Healthoutmed.RData")

jpeg(file="Results/CM_healthout_b2.jpeg")
plot1 <- plot(healthout, xlab = "Effect Size")
legend("topright", inset=.02, legend = c("Education up to 16","Education after 16"),
       lty = c(1,2),
       pch = c(16,1),
       box.lty=0)
title("b) Mediation effects", adj = 0)
mtext("ACME: Average causal mediation effect\nADE: Average direct effect\n", side = 3, adj=1)

dev.off()

# Coefficients taken from amelidiate output
data <- c(0, "'0.25'", 0,
          0, 0, 0,
          "'0.15'", "'0.03'", 0)
M<- matrix (nrow=3, ncol=3, byrow = TRUE, data=data)

jpeg(file="Results/CM_healthout_a2.jpeg")

plot2 <- plotmat(M, pos=c(1,2),
                 name= c( "Health Outcome","Education", "Dementia"),
                 box.type = "rect", box.size = 0.18, box.prop=0.3,  curve=0,
                 shadow.size = 0)

title("a) Mediation paths", adj = 0)

mtext("Path coefficient scores provided on paths", side = 3, adj=0)

dev.off()

## ---------- Income ---------------------------------------------------------------------------

data <- names(completemice)

income <- lapply(completemice, FUN = function(data){

  out <- list()
  count <- 1

  ind <- glm(income_dum ~ edu_dum + sexc + age + ethnicity_cat_c, data = data,
             family=binomial(link="probit"))
  dir <- glm(dem_dum ~ edu_dum + income_dum + sexc + age + ethnicity_cat_c + healthb_dum + healtho_dum, data = data,
             family=binomial(link="probit"))
  out <- mediate(ind, dir, sims=1000, treat="edu_dum",mediator="income_dum")
  summary(out)
  class(out) <- "mediations"
  return(out)
  # out
})


names <- letters[1:20]
names(income) <- names

income2 <- amelidiate(income)

saveRDS(income2, file = "Results/income.RData")

summary(income2)
plot(income2)

jpeg(file="Results/CM_income.jpeg")
plot(income2)
dev.off()

#income <- readRDS("Results/income.RData")

jpeg(file="Results/CM_income_b2.jpeg")
plot1 <- plot(income, xlab = "Effect Size")
legend("topright", inset=.02, legend = c("Education up to 16","Education after 16"),
       lty = c(1,2),
       pch = c(16,1),
       box.lty=0)
title("b) Mediation effects", adj = 0)
mtext("ACME: Average causal mediation effect\nADE: Average direct effect\n", side = 3, adj=1)

dev.off()


data <- c(0, "'0.49'", 0,
          0, 0, 0,
          "'0.05'", "'0.09'", 0)
M<- matrix (nrow=3, ncol=3, byrow = TRUE, data=data)

jpeg(file="Results/CM_income_a2.jpeg")

plot2 <- plotmat(M, pos=c(1,2),
                 name= c( "Income","Education", "Dementia"),
                 box.type = "rect", box.size = 0.18, box.prop=0.3,  curve=0,
                 shadow.size = 0)

title("a) Mediation paths", adj = 0)
mtext("Path coefficient scores provided on paths", side = 3, adj=0)

dev.off()


## --------- Occupation -----------------------------------------------------------------------------

data <- names(completemice)

occupation <- lapply(completemice, FUN = function(data){
```

```
  out <- list()
  count <- 1

  ind <- glm(occupationb_dum ~ edu_dum + sexc + age + ethnicity_cat_c, data = data,
             family=binomial(link="probit"))
  dir <- glm(dem_dum ~ edu_dum + occupationb_dum + sexc + age + ethnicity_cat_c + healthb_dum + healtho_dum, data = data,
             family=binomial(link="probit"))
  out <- mediate(ind, dir, sims=1000, treat="edu_dum",mediator="occupationb_dum")
  summary(out)
  class(out) <- "mediations"
  return(out)
  # out
})


names <- letters[1:20]
names(occupation) <- names

occupation2 <- amelidiate(occupation)

summary(occupation2)

jpeg(file="Results/CM_occupation_b2.jpeg")
plot1 <- plot(occupation2, xlab = "Effect Size")
legend("topright", inset=.02, legend = c("Education up to 16","Education after 16"),
       lty = c(1,2),
       pch = c(16,1),
       box.lty=0)
title("b) Mediation effects", adj = 0)
mtext("ACME: Average causal mediation effect\nADE: Average direct effect\n", side = 3, adj=1)

dev.off()

data <- c(0, "'0.28'", 0,
          0, 0, 0,
          "'0.03'", "'0.95'", 0)
M<- matrix (nrow=3, ncol=3, byrow = TRUE, data=data)

Cairo::Cairo(file="Results/CM_occupation_a2.png",
             width = 170,
             height = 170,
             units = "mm",
             dpi=300,
             bg = "white",
             pointsize = 10)
plot2 <- plotmat(M, pos=c(1,2),
                 name= c( "Occupational Complexity","Education", "Dementia"),
                 box.type = "rect", box.size = 0.18, box.prop=0.3,  curve=0,
                 shadow.size = 0)

title("a) Mediation paths", adj = 0)
mtext("Path coefficient scores provided on paths", side = 3, adj=0)

dev.off()

Cairo::Cairo(file="Results/CM_occupation_b2.png",
             width = 170,
             height = 170,
             units = "mm",
             dpi=300,
             bg = "white",
             pointsize = 10)
plot1 <- plot(occupation2, xlab = "Effect Size")
legend("topright", inset=.02, legend = c("Education up to 16","Education after 16"),
       lty = c(1,2),
       pch = c(16,1),
       box.lty=0)
title("b) Mediation effects", adj = 0)
mtext("ACME: Average causal mediation effect\nADE: Average direct effect\n", side = 3, adj=1)

dev.off()

#png(file="Results/CM_occupation_a.png", res = 300)


## ---------- Social isolation -------------------------------------------------------------------

data <- names(completemice)

isolation <- lapply(completemice, FUN = function(data){

  out <- list()
  count <- 1

  ind <- glm(socialb_dum ~ edu_dum + sexc + age + ethnicity_cat_c, data = data,
             family=binomial(link="probit"))
  dir <- glm(dem_dum ~ edu_dum + socialb_dum + sexc + age + ethnicity_cat_c + depression2 , data = data,
             family=binomial(link="probit"))
  out <- mediate(ind, dir, sims=1000, treat="edu_dum",mediator="socialb_dum")
  summary(out)
  class(out) <- "mediations"
  return(out)
  # out
})


names <- letters[1:20]
names(isolation) <- names

isolation2 <- amelidiate(isolation)

summary(isolation2)
plot(isolation2)
```

```
saveRDS(isolation2, file = "Results/isolation.RData")

jpeg(file="Results/CM_isolation.jpeg")
plot(isolation2)
dev.off()

#isolation <- readRDS("Results/isolation.RData")
#summary(isolation)

## ----------- Health Behaviours --------------------


data <- names(completemice)

healthbehav <- lapply(completemice, FUN = function(data){

  out <- list()
  count <- 1

  ind <- glm(healthb_dum ~ edu_dum + sexc + age + ethnicity_cat_c, data = data,
             family=binomial(link="probit"))
  dir <- glm(dem_dum ~ edu_dum + healthb_dum + sexc + age + ethnicity_cat_c + healtho_dum + income_dum + occupationb_dum, data =
data,
             family=binomial(link="probit"))
  out <- mediate(ind, dir, sims=1000, treat="edu_dum",mediator="healthb_dum")
  summary(out)
  class(out) <- "mediations"
  return(out)
  # out
})


names <- letters[1:20]
names(healthbehav) <- names

healthbehav2 <- amelidiate(healthbehav)
saveRDS(healthbehav2, file = "Results/Healthbehav.RData")

healthbehav <- readRDS("Results/Healthbehav.RData")
summary(healthbehav)

plot(healthbehav)

jpeg(file="Results/CM_healthbehav.jpeg")
plot(healthbehav)
dev.off()

jpeg(file="Results/CM_healthbehav_b2.jpeg")
plot1 <- plot(healthbehav, xlab = "Effect Size")
legend("topright", inset=.02, legend = c("Education up to 16","Education after 16"),
       lty = c(1,2),
       pch = c(16,1),
       box.lty=0)
title("b) Mediation effects", adj = 0)
mtext("ACME: Average causal mediation effect\nADE: Average direct effect\n", side = 3, adj=1)

dev.off()


data <- c(0, "'0.38'", 0,
          0, 0, 0,
          "'0.03'", "'0.13'", 0)
M<- matrix (nrow=3, ncol=3, byrow = TRUE, data=data)

jpeg(file="Results/CM_healthbehav_a2.jpeg")

plot2 <- plotmat(M, pos=c(1,2),
                 name= c( "Health Behaviour","Education", "Dementia"),
                 box.type = "rect", box.size = 0.18, box.prop=0.3,  curve=0,
                 shadow.size = 0)

title("a) Mediation paths", adj = 0)
mtext("Path coefficient scores provided on paths", side = 3, adj=0)

dev.off()


## ----------- Basic graph -------------------------------------------------------

data <- c(0, "a", 0,
          0, 0, 0,
          "b", "c", 0)
M<- matrix (nrow=3, ncol=3, byrow = TRUE, data=data)

Cairo::Cairo(file="Results/basic_model2.png",
             width = 170,
             height = 170,
             units = "mm",
             dpi=300,
             bg = "white",
             pointsize = 10)
plot2 <- plotmat(M, pos=c(1,2),
                 name= c( "Mediator","Education", "Dementia"),
                 box.type = "rect", box.size = 0.18, box.prop=0.3,  curve=0,
                 shadow.size = 0)

dev.off()

## ----------- Combined occupation figure -------------------------------------------

Cairo::Cairo(file="Results/CM_occupation_final2.png",
             width = 170,
             height = 350,
```

```
            units = "mm",
            dpi=300,
            bg = "white",
            pointsize = 10)
par(mfrow=c(2,1))

plot2 <- plotmat(M, pos=c(1,2),
                 name= c( "Occupational Complexity","Education", "Dementia"),
                 box.type = "rect", box.size = 0.18, box.prop=0.3,  curve=0,
                 shadow.size = 0)

title("a) Mediation paths", adj = 0)
mtext("Path coefficient scores provided on paths", side = 3, adj=0)


plot1 <- plot(occupation2, xlab = "Effect Size")
legend("topright", inset=.02, legend = c("Education up to 16","Education after 16"),
       lty = c(1,2),
       pch = c(16,1),
       box.lty=0)
title("b) Mediation effects", adj = 0)
mtext("ACME: Average causal mediation effect\nADE: Average direct effect\n", side = 3, adj=1)

dev.off()



# Mediation analysis - complete case analysis - (Code 9) ##################################################

## ----------- Set up -------------------------------------------------------------------------------

library(tidyverse)
library(mice)
library(mitools)
library(norm)
library("writexl")
library(VIM)
library(miceadds)
library(lavaan)
library(mitml)
library(semPlot)
library(intmed)
library(mediation)
library(diagram)
library(ggplot2)
library(tidySEM)

setwd("/Volumes/GroupFolders/MHS_Naaheed_UKB/Katherine Taylor")
load("complete_case_data_fix.Rdata")

complete_case_data_fix$alldem_c <- factor(complete_case_data_fix$alldem_c, order = TRUE, levels =c('No dementia', 'Dementia'))

complete_case_data_fix <- complete_case_data_fix %>%
  mutate(dem_dummy = case_when(alldem_c == 'No dementia' ~ 0,
                               alldem_c == 'Dementia' ~ 1))

qcheck <- table(complete_case_data_fix$dem_dummy)
barplot(qcheck)

complete_case_data_fix$edu_cat_b <- as.factor(complete_case_data_fix$edu_cat_b)

complete_case_data_fix$edu_dum <- case_when(complete_case_data_fix$edu_cat_b == "Education up 16" ~ 1,
                                            TRUE ~ 0)

#Set mediators as binary 0/1

complete_case_data_fix$income_dum <- case_when(complete_case_data_fix$income_b == "< 31,000" ~ 1,
                                               TRUE ~ 0 )
complete_case_data_fix$healtho_dum <- case_when(complete_case_data_fix$ho_summary_b == "Intermediate/Poor health" ~ 1,
                                                TRUE ~ 0)
complete_case_data_fix$healthb_dum <- case_when(complete_case_data_fix$hb_summary_b == "Unfavourable" ~ 1,
                                                TRUE ~ 0)
complete_case_data_fix$occupationb_dum <- case_when(complete_case_data_fix$occupation_b == "Non mangerial or proffesional
occupations" ~ 1,
                                                    TRUE ~ 0)

complete_case_data_fix$socialb_dum <- case_when(complete_case_data_fix$social_c == "Socially isolated" ~ 1,
                                                TRUE ~ 0)


complete_case_data_fix2 <- complete_case_data_fix %>%
  dplyr::select(1,2,4,17,20:27)


qcheck <- table(complete_case_data_fix2$edu_dum)
barplot(qcheck)


## ----------- Health Behaviour as a mediator -------------------------------------------------------

ind <- glm(healthb_dum ~ edu_dum + sexc + age + ethnicity_cat_c, data = complete_case_data_fix2,
           family=binomial(link="probit"))
dir <- glm(dem_dummy ~ edu_dum +healthb_dum + sexc + age + ethnicity_cat_c + healthb_dum + income_dum + occupationb_dum, data =
complete_case_data_fix2,
           family=binomial(link="probit"))
out <- mediate(ind, dir, sims=1000, treat="edu_dum",mediator="healthb_dum")
summary(out)


## Health Outcome as a mediator ---------------------------------------------------------------------

ind <- glm(healtho_dum ~ edu_dum + sexc + age + ethnicity_cat_c, data = complete_case_data_fix2,
           family=binomial(link="probit"))
dir <- glm(dem_dummy ~ edu_dum + healtho_dum + sexc + age + ethnicity_cat_c + healthb_dum + income_dum + occupationb_dum, data =
```

```
complete_case_data_fix2,
           family=binomial(link="probit"))
out <- mediation::mediate(ind, dir, sims=1000, treat="edu_dum", mediator="healtho_dum")
summary(out)

saveRDS(out, file = "Results/Healthoutmed_cc.RData")


jpeg(file="Results/CM_healthoutcome_cc.jpeg")
plot(out)
dev.off()


## ----------- Income as mediator -------------------------------------------------------------------

ind <- glm(income_dum ~ edu_dum + sexc + age + ethnicity_cat_c, data = complete_case_data_fix2,
           family=binomial(link="probit"))
dir <- glm(dem_dummy ~ edu_dum + income_dum + sexc + age + ethnicity_cat_c + healthb_dum + healtho_dum, data =
complete_case_data_fix2,
           family=binomial(link="probit"))
out <- mediation::mediate(ind, dir, sims=1000, treat="edu_dum",mediator="income_dum")
summary(out)


saveRDS(out, file = "Results/Incomemed_cc.RData")


jpeg(file="Results/CM_income_cc.jpeg")
plot(out)
dev.off()

## ----------- Occupational Complexity as mediator -------------------------------------------------------

ind <- glm(occupationb_dum ~ edu_dum + sexc + age + ethnicity_cat_c, data = complete_case_data_fix2,
           family=binomial(link="probit"))
dir <- glm(dem_dummy ~ edu_dum + occupationb_dum + sexc + age + ethnicity_cat_c + healthb_dum + healtho_dum, data =
complete_case_data_fix2,
           family=binomial(link="probit"))
out <- mediation::mediate(ind, dir, sims=1000, treat="edu_dum",mediator="occupationb_dum")
summary(out)

saveRDS(out, file = "Results/Occcomplexmed_cc.RData")

jpeg(file="Results/CM_occupation_cc.jpeg")
plot(out)
dev.off()


## ----------- Social Isolation as mediator -------------------------------------------------------------------

ind <- glm(socialb_dum ~ edu_dum + sexc + age + ethnicity_cat_c, data = complete_case_data_fix2,
           family=binomial(link="probit"))
dir <- glm(dem_dummy ~ edu_dum + socialb_dum + sexc + age + ethnicity_cat_c + depression2 , data = complete_case_data_fix2,
           family=binomial(link="probit"))
out <- mediation::mediate(ind, dir, sims=1000, treat="edu_dum",mediator="socialb_dum")
summary(out)


# Mediation analysis - testing for interactions - (Code 10) ###########################################

## ----------- Set up ------------------------------------------------------------------

setwd("/Volumes/GroupFolders/MHS_Naaheed_UKB/Katherine Taylor")
library(mice)
library(tidyverse)
library(mediation)
library(mitools)
library(miceadds)
library(diagram)

install.packages("Cairo")
library("Cairo")

load("imp_long_fix.RData")

imp_long3$edu_cat_b <- as.factor(imp_long3$edu_cat_b)

imp_long3$edu_dum <- case_when(imp_long3$edu_cat_b == "Education up 16" ~ 1,
                               TRUE ~ 0)

imp_long3$dem_dum <- case_when(imp_long3$alldem_c == "Dementia" ~ 1,
                               TRUE ~ 0)


#Set mediators as binary 0/1

imp_long3$income_dum <- case_when(imp_long3$income_b == "< 31,000" ~ 1,
                                  TRUE ~ 0)
imp_long3$healtho_dum <- case_when(imp_long3$ho_summary_b == "Intermediate/Poor health" ~ 1,
                                   TRUE ~ 0)
imp_long3$healthb_dum <- case_when(imp_long3$hb_summary_b == "Unfavourable" ~ 1,
                                   TRUE ~ 0)
imp_long3$occupationb_dum <- case_when(imp_long3$occupation_b == "Non mangerial or proffesional occupations" ~ 1,
                                       TRUE ~ 0)

imp_long3$socialb_dum <- case_when(imp_long3$social_c == "Socially isolated" ~ 1,
                                   TRUE ~ 0)


imp_long4 <- imp_long3 %>%
  dplyr::select(1,2,4,6,19,21:29)

imp_test <-as.mids(imp_long4)
```

```
completemice <- imp_test %>%
  mice::complete("all")

# Testing for interactions

##  ----------- Health Outcomes -----------------------------------------------------------------

# Run mediation models with interaction term for each imputation and perform a significance
# test (Tmint). Save outputs in a list. Apply Rubins rules to get an average coefficient and
# 95% CI for significance test. Use ammelidate function to observe proportion mediated.

### ==== Mediation analysis with interaction term ====================================

healthoutcome <- lapply(completemice, FUN = function(data){

  ind <- glm(healtho_dum ~ edu_dum + sexc + age + ethnicity_cat_c, data = data,
             family=binomial(link="probit"))
  dir <- glm(dem_dum ~ edu_dum*healtho_dum + sexc + age + ethnicity_cat_c + healthb_dum + income_dum + occupationb_dum, data = data,
             family=binomial(link="probit"))
  out <- mediate(ind, dir, sims=1000, treat="edu_dum",mediator="healtho_dum")
  Tmint <- test.TMint(out)

  summary(out)

  count <- 1
  #out <- list()
  class(out) <- "mediations"
  #return(out)

  return(list(out=out, Tmint =Tmint))

  # out
})

### ==== Get the average interaction statistic and 95% CI ============

## CI
healthoutcome_ci <- lapply(healthoutcome, FUN = function(data){
  out <- data$Tmint$conf.int
  return(out)
})

# Flatten the list of CIs
all_cis <- do.call(rbind, healthoutcome_ci)

# Calculate the average lower bound of the CIs
average_lower <- mean(all_cis[, 1])

# Calculate the average upper bound of the CIs
average_upper <- mean(all_cis[, 2])

# Print the average CI
cat("Average CI: [", average_lower, ",", average_upper, "]\n")

## Mean

healthoutcome_mean <- lapply(healthoutcome, FUN = function(data){
  out <- data$Tmint$statistic
  return(out)
})

# Flatten the list of CIs
all_m <- do.call(rbind, healthoutcome_mean)

# Calculate the average lower bound of the CIs
average_mean <- mean(all_m)

# Print the average CI
cat("Average statistic: [", average_mean, "]\n")


### ==== Check ACME, ADE and proportion mediated =================

healthoutcome_int <- lapply(healthoutcome, FUN = function(data){
  out <- data$out
  return(out)})

names <- letters[1:20]
names(healthoutcome_int) <- names

healthoutcome_int2 <- amelidiate(healthoutcome_int)
summary(healthoutcome_int2)

saveRDS(healthoutcome_int, file = "Results/healthoutcome_int.RData")

## ----------- Occupational Complexity --------------------------------------------

### ==== Mediation analysis with interaction term ====================================

occupation <- lapply(completemice, FUN = function(data){

  ind <- glm(occupationb_dum ~ edu_dum + sexc + age + ethnicity_cat_c, data = data,
             family=binomial(link="probit"))
  dir <- glm(dem_dum ~ edu_dum*occupationb_dum + sexc + age + ethnicity_cat_c + healthb_dum + healtho_dum, data = data,
             family=binomial(link="probit"))
  out <- mediate(ind, dir, sims=1000, treat="edu_dum",mediator="occupationb_dum")
  Tmint <- test.TMint(out)

  summary(out)

  count <- 1
  #out <- list()
  class(out) <- "mediations"
  #return(out)
```

```
  return(list(out=out, Tmint =Tmint))

  # out
})

### ==== Get the average interaction statistic and 95% CI ============


# Get the mean difference in ACME and average CI

## CI
occupation_ci <- lapply(occupation, FUN = function(data){
  out <- data$Tmint$conf.int
  return(out)
})

# Flatten the list of CIs
all_occupation_cis <- do.call(rbind, occupation_ci)

# Calculate the average lower bound of the CIs
average_lower_occupation <- mean(all_occupation_cis[, 1])

# Calculate the average upper bound of the CIs
average_upper_occupation <- mean(all_occupation_cis[, 2])

# Print the average CI
cat("Average CI: [", average_lower_occupation, ",", average_upper_occupation, "]\n")

## Mean

occupation_mean <- lapply(occupation, FUN = function(data){
  out <- data$Tmint$statistic
  return(out)
})

# Flatten the list of CIs
all_m_occupation <- do.call(rbind, occupation_mean)

# Calculate the average lower bound of the CIs
average_mean_occupation <- mean(all_m_occupation)

# Print the average CI
cat("Average statistic: [", average_mean_occupation, "]\n")


### ==== Check ACME, ADE and proportion mediated =================

occupation_int <- lapply(occupation, FUN = function(data){
  out <- data$out
  return(out)})

names <- letters[1:20]
names(occupation_int) <- names

occupation_int2 <- amelidiate(occupation_int)
summary(occupation_int2)

saveRDS(occupation_int2, file = "Results/occupation_int.RData")


## -------- Income ---------------------------------------------------------------

### ==== Mediation analysis with interaction term ===================================

income <- lapply(completemice, FUN = function(data){


  ind <- glm(income_dum ~ edu_dum + sexc + age + ethnicity_cat_c, data = data,
             family=binomial(link="probit"))
  dir <- glm(dem_dum ~ edu_dum*income_dum + sexc + age + ethnicity_cat_c + healthb_dum + healtho_dum, data = data,
             family=binomial(link="probit"))
  out <- mediate(ind, dir, sims=1000, treat="edu_dum",mediator="income_dum")
  Tmint <- test.TMint(out)

  summary(out)

  count <- 1
  #out <- list()
  class(out) <- "mediations"
  #return(out)

  return(list(out=out, Tmint =Tmint))

  # out
})

### ==== Get the average interaction statistic and 95% CI ============

# Get the mean difference in ACME and average CI

## CI
income_ci <- lapply(income, FUN = function(data){
  out <- data$Tmint$conf.int
  return(out)
})

# Flatten the list of CIs
all_cis_income <- do.call(rbind, income_ci)

# Calculate the average lower bound of the CIs
average_lower_income <- mean(all_cis_income[, 1])

# Calculate the average upper bound of the CIs
```

```
average_upper_income <- mean(all_cis_income[, 2])

# Print the average CI
cat("Average CI: [", average_lower_income, ",", average_upper_income, "]\n")

## Mean

income_mean <- lapply(income, FUN = function(data){
  out <- data$Tmint$statistic
  return(out)
})

# Flatten the list of CIs
all_m_income <- do.call(rbind, income_mean)

# Calculate the average lower bound of the CIs
average_mean_income <- mean(all_m_income)

# Print the average CI
cat("Average statistica: [", average_mean_income, "]\n")

### ==== Check ACME, ADE and proportion mediated =================

income_int <- lapply(income, FUN = function(data){
  out <- data$out
  return(out)})

names <- letters[1:20]
names(income_int) <- names

income_int2 <- amelidiate(income_int)
summary(income_int2)

saveRDS(income_int2, file = "Results/income_int.RData")


## -------- Health behaviours ----------------------------------------

### ==== Mediation analysis with interaction term ===================================

healthbehaviours <- lapply(completemice, FUN = function(data){

  ind <- glm(healthb_dum ~ edu_dum + sexc + age + ethnicity_cat_c, data = data,
             family=binomial(link="probit"))
  dir <- glm(dem_dum ~ edu_dum*healthb_dum + sexc + age + ethnicity_cat_c + healtho_dum + income_dum + occupationb_dum, data = data,
             family=binomial(link="probit"))
  out <- mediate(ind, dir, sims=1000, treat="edu_dum",mediator="healthb_dum")
  Tmint <- test.TMint(out)

  summary(out)

  count <- 1
  #out <- list()
  class(out) <- "mediations"
  #return(out)

  return(list(out=out, Tmint =Tmint))

  # out
})

### ==== Get the average interaction statistic and 95% CI ============

# Get the mean difference in ACME and average CI

## CI
healthbehaviours_ci <- lapply(healthbehaviours, FUN = function(data){
  out <- data$Tmint$conf.int
  return(out)
})

# Flatten the list of CIs
all_cis_healthbehaviours <- do.call(rbind, healthbehaviours_ci)

# Calculate the average lower bound of the CIs
average_lower_hb <- mean(all_cis_healthbehaviours[, 1])

# Calculate the average upper bound of the CIs
average_upper_hb <- mean(all_cis_healthbehaviours[, 2])

# Print the average CI
cat("Average CI: [", average_lower_hb, ",", average_upper_hb, "]\n")

## Mean

healthbehav_mean <- lapply(healthbehaviours, FUN = function(data){
  out <- data$Tmint$statistic
  return(out)
})

# Flatten the list of CIs
all_m_hb <- do.call(rbind, healthbehav_mean)

# Calculate the average lower bound of the CIs
average_mean_hb <- mean(all_m_hb)

# Print the average CI
cat("Average statistica: [", average_mean_hb, "]\n")


### ==== Check ACME, ADE and proportion mediated =================

healthbehav_int <- lapply(healthbehaviours, FUN = function(data){
  out <- data$out
```

```
  return(out)})

names <- letters[1:20]
names(healthbehav_int) <- names

healthbehav_int2 <- amelidiate(healthbehav_int)
summary(healthbehav_int2)

saveRDS(healthbehav_int2, file = "Results/healthbehav_int.RData")


## ---------- Income -------------------------------------------------------------

### ==== Mediation analysis with interaction term ===================================

income <- lapply(completemice, FUN = function(data){


  ind <- glm(income_dum ~ edu_dum + sexc + age + ethnicity_cat_c, data = data,
             family=binomial(link="probit"))
  dir <- glm(dem_dum ~ edu_dum*income_dum + sexc + age + ethnicity_cat_c + healthb_dum + healtho_dum, data = data,
             family=binomial(link="probit"))
  out <- mediate(ind, dir, sims=1000, treat="edu_dum",mediator="income_dum")
  Tmint <- test.TMint(out)

  summary(out)

  count <- 1
  #out <- list()
  class(out) <- "mediations"
  #return(out)

  return(list(out=out, Tmint =Tmint))

  # out
})

### ==== Get the average interaction statistic and 95% CI ============

# Get the mean difference in ACME and average CI

## CI
income_ci <- lapply(income, FUN = function(data){
  out <- data$Tmint$conf.int
  return(out)
})

# Flatten the list of CIs
all_cis_income <- do.call(rbind, income_ci)

# Calculate the average lower bound of the CIs
average_lower_income <- mean(all_cis_income[, 1])

# Calculate the average upper bound of the CIs
average_upper_income <- mean(all_cis_income[, 2])

# Print the average CI
cat("Average CI: [", average_lower_income, ",", average_upper_income, "]\n")

## Mean

income_mean <- lapply(income, FUN = function(data){
  out <- data$Tmint$statistic
  return(out)
})

# Flatten the list of CIs
all_m_income <- do.call(rbind, income_mean)

# Calculate the average lower bound of the CIs
average_mean_income <- mean(all_m_income)

# Print the average CI
cat("Average statistica: [", average_mean_income, "]\n")

### ==== Check ACME, ADE and proportion mediated =================

income_int <- lapply(income, FUN = function(data){
  out <- data$out
  return(out)})

names <- letters[1:20]
names(income_int) <- names

income_int2 <- amelidiate(income_int)
summary(income_int2)

saveRDS(income_int2, file = "Results/income_int.RData")

## --------- Social isolation ---------------------------------------------

### ==== Mediation analysis with interaction term ===================================

socialisolation <- lapply(completemice, FUN = function(data){

  ind <- glm(socialb_dum ~ edu_dum + sexc + age + ethnicity_cat_c, data = data,
             family=binomial(link="probit"))
  dir <- glm(dem_dum ~ edu_dum*socialb_dum + sexc + age + ethnicity_cat_c + depression2 , data = data,
             family=binomial(link="probit"))
  out <- mediate(ind, dir, sims=1000, treat="edu_dum",mediator="socialb_dum")
  Tmint <- test.TMint(out)

  summary(out)
```

```
  count <- 1
  #out <- list()
  class(out) <- "mediations"
  #return(out)

  return(list(out=out, Tmint =Tmint))

  # out
})

### ==== Get the average interaction statistic and 95% CI ============

# Get the mean difference in ACME and average CI

## CI
socialisolation_ci <- lapply(socialisolation, FUN = function(data){
  out <- data$Tmint$conf.int
  return(out)
})

# Flatten the list of CIs
all_cis_socialisolation <- do.call(rbind, socialisolation_ci)

# Calculate the average lower bound of the CIs
average_lower_si <- mean(all_cis_socialisolation[, 1])

# Calculate the average upper bound of the CIs
average_upper_si <- mean(all_cis_socialisolation[, 2])

# Print the average CI
cat("Average CI: [", average_lower_si, ",", average_upper_si, "]\n")

## Mean

socialiso_mean <- lapply(socialisolation, FUN = function(data){
  out <- data$Tmint$statistic
  return(out)
})

# Flatten the list of CIs
all_m_si <- do.call(rbind, socialiso_mean)

# Calculate the average lower bound of the CIs
average_mean_si <- mean(all_m_si)

# Print the average CI
cat("Average statistica: [", average_mean_si, "]\n")

### ==== Check ACME, ADE and proportion mediated =================

socialiso_int <- lapply(socialisolation, FUN = function(data){
  out <- data$out
  return(out)})

names <- letters[1:20]
names(socialiso_int) <- names

socialiso_int2 <- amelidiate(socialiso_int)
summary(socialiso_int2)


# Mediation analysis - testing for interactions, complete case analysis - (Code 11) ############################################

## ----------- Set up ---------------------------------------------------------------------------

library(tidyverse)
library(mice)
library(mitools)
library(norm)
library("writexl")
library(VIM)
library(miceadds)
library(lavaan)
library(mitml)
library(semPlot)
library(intmed)
library(mediation)
library(diagram)
library(ggplot2)
library(tidySEM)

setwd("/Volumes/GroupFolders/MHS_Naaheed_UKB/Katherine Taylor")
load("complete_case_data_fix.Rdata")

complete_case_data_fix$alldem_c <- factor(complete_case_data_fix$alldem_c, order = TRUE, levels =c('No dementia', 'Dementia'))

complete_case_data_fix <- complete_case_data_fix %>%
  mutate(dem_dummy = case_when(alldem_c == 'No dementia' ~ 0,
                               alldem_c == 'Dementia' ~ 1))

qcheck <- table(complete_case_data_fix$dem_dummy)
barplot(qcheck)

complete_case_data_fix$edu_cat_b <- as.factor(complete_case_data_fix$edu_cat_b)

complete_case_data_fix$edu_dum <- case_when(complete_case_data_fix$edu_cat_b == "Education up 16" ~ 1,
                                            TRUE ~ 0)

#Set mediators as binary 0/1

complete_case_data_fix$income_dum <- case_when(complete_case_data_fix$income_b == "< 31,000" ~ 1,
                                               TRUE ~ 0 )
complete_case_data_fix$healtho_dum <- case_when(complete_case_data_fix$ho_summary_b == "Intermediate/Poor health" ~ 1,
                                                TRUE ~ 0)
```

```
complete_case_data_fix$healthb_dum <- case_when(complete_case_data_fix$hb_summary_b == "Unfavourable" ~ 1,
                                                TRUE ~ 0)
complete_case_data_fix$occupationb_dum <- case_when(complete_case_data_fix$occupation_b == "Non mangerial or proffesional
occupations" ~ 1,
                                                    TRUE ~ 0)

complete_case_data_fix$socialb_dum <- case_when(complete_case_data_fix$social_c == "Socially isolated" ~ 1,
                                                TRUE ~ 0)


complete_case_data_fix2 <- complete_case_data_fix %>%
  dplyr::select(1,2,4,17,20:27)


qcheck <- table(complete_case_data_fix2$edu_dum)
barplot(qcheck)



## -------- Health behaviour ------------------------------------------------------------------------

ind <- glm(healthb_dum ~ edu_dum + sexc + age + ethnicity_cat_c, data = complete_case_data_fix2,
           family=binomial(link="probit"))
dir <- glm(dem_dummy ~ edu_dum*healthb_dum + sexc + age + ethnicity_cat_c + healthb_dum + income_dum + occupationb_dum, data =
complete_case_data_fix2,
           family=binomial(link="probit"))
out <- mediate(ind, dir, sims=1000, treat="edu_dum",mediator="healthb_dum")

healthbehav <- out
summary(healthbehav)
test.TMint(healthbehav)

## -------- Health outcome --------------------------------------------------------------------------

ind <- glm(healtho_dum ~ edu_dum + sexc + age + ethnicity_cat_c, data = complete_case_data_fix2,
           family=binomial(link="probit"))
dir <- glm(dem_dummy ~ edu_dum*healtho_dum + sexc + age + ethnicity_cat_c + healthb_dum + income_dum + occupationb_dum, data =
complete_case_data_fix2,
           family=binomial(link="probit"))
out <- mediation::mediate(ind, dir, sims=1000, treat="edu_dum", mediator="healtho_dum")


healthout <- out
summary(healthout)
test.TMint(healthout)

## -------- Income ----------------------------------------------------------------------------------

ind <- glm(income_dum ~ edu_dum + sexc + age + ethnicity_cat_c, data = complete_case_data_fix2,
           family=binomial(link="probit"))
dir <- glm(dem_dummy ~ edu_dum*income_dum + sexc + age + ethnicity_cat_c + healthb_dum + healtho_dum, data = complete_case_data_fix2,
           family=binomial(link="probit"))
out <- mediation::mediate(ind, dir, sims=1000, treat="edu_dum",mediator="income_dum")


income <- out
summary(income)
test.TMint(income)

## -------- Occupational complexity --------------------------------------------------------

ind <- glm(occupationb_dum ~ edu_dum + sexc + age + ethnicity_cat_c, data = complete_case_data_fix2,
           family=binomial(link="probit"))
dir <- glm(dem_dummy ~ edu_dum*occupationb_dum + sexc + age + ethnicity_cat_c + healthb_dum + healtho_dum, data =
complete_case_data_fix2,
           family=binomial(link="probit"))
out <- mediation::mediate(ind, dir, sims=1000, treat="edu_dum",mediator="occupationb_dum")


occupationalc <- out
summary(occupationalc)
test.TMint(occupationalc)

## -------- Social Isolation ------------------------------------------------------------------------

ind <- glm(socialb_dum ~ edu_dum + sexc + age + ethnicity_cat_c, data = complete_case_data_fix2,
           family=binomial(link="probit"))
dir <- glm(dem_dummy ~ edu_dum*socialb_dum + sexc + age + ethnicity_cat_c + depression2 , data = complete_case_data_fix2,
           family=binomial(link="probit"))
out <- mediation::mediate(ind, dir, sims=1000, treat="edu_dum",mediator="socialb_dum")
summary(out)


socialiso <- out
summary(socialiso)
test.TMint(socialiso)
```
